# Transcriptomic Insights into Late-Life Depression and the Role of Environmental Drinking Water Composition: A Study on 18-Month-Old Mice

**DOI:** 10.3390/ijms262110626

**Published:** 2025-10-31

**Authors:** João Pedro Costa-Nunes, Kseniia Sitdikova, Evgeniy Svirin, Johannes de Munter, Gabor Somlyai, Anna Gorlova, Alexandr Litavrin, Gohar M. Arajyan, Zlata Nefedova, Alexei Lyundup, Sergey Morozov, Aleksei Umriukhin, Sofia Iliynskaya, Anton Chernopiatko, Tatyana Strekalova

**Affiliations:** 1 Faculdade de Medicina, Universidade de Lisboa, Campo Grande, 1749-016 Lisboa, Portugal; jpcosta.nunes@gmail.com; 2 Department of Psychiatry and Neuropsychology, Maastricht University, 6229 ER Maastricht, The Netherlandsh.demunter@neuroplast.com (J.d.M.); anna.gorlova204@gmail.com (A.G.); 3 Institute of General Pathology and Pathophysiology, Russian Academy of Medical Sciences, 125315 Moscow, Russia; sitdikova.k@niiopp.ru (K.S.);; 4 Neuroplast B.V., 6222 NK Maastricht, The Netherlands; 5 HYD LLC for Cancer Research and Drug Development, 1118 Budapest, Hungary; gsomlyai@hyd.hu; 6 Research and Education Resource Center, Peoples Friendship University of Russia (RUDN University), 117198 Moscow, Russia; liundup_av@pfur.ru; 7 Department of Normal Physiology, Sechenov University, 119991 Moscow, Russia; alexlitavrin29@gmail.com (A.L.); zlanefedova@gmail.com (Z.N.); alum1@yandex.ru (A.U.); 8 Pharmacology and Pathohistology Laboratory, Scientific Technological Center of the Organic and Pharmaceutical Chemistry Institute of Armenia, Yerevan 0019, Armenia; arajyankens@gmail.com; 9 Timantti AB, 174 07 Stockholm, Swedenchernopiatko@gmail.com (A.C.)

**Keywords:** late-life depression, aging, anhedonia, depression, environment, deuterium, Illumina gene profiling, animal models, mice

## Abstract

The study of molecular mechanisms underlying late-life depression (LLD) is increasingly important in light of population aging. To date, LLD-related molecular brain changes remain poorly understood. Furthermore, environmental factors such as climate change and geography contribute to LDD risks. One overlooked factor might be deuterium—a stable hydrogen isotope—whose concentration in drinking water can vary geographically (~90–155 ppm) and alter the incidence of mood disorders. Conversely, potential effects of natural variations in deuterium content in drinking water on LLD symptoms and brain gene expression remain unknown. We conducted Illumina gene expression profiling in the hippocampi and prefrontal cortexes of 18-month-old C57BL/6J mice, a model of LLD-like behaviors, compared to 3-month-old controls. Separately, aged mice were allowed to consume deuterium-depleted (DDW, ~90 ppm) or control (~140 ppm) water for 21 days and were studied for LLD-like behaviors and Illumina gene expression of the brain. Naïve old mice displayed ≥2-fold significant changes of 35 genes. Housing on DDW increased their hedonic sensitivity and novelty exploration, reduced helplessness, improved memory, and significantly altered brain expression of *Egr1*, *Per2*, *Homer1*, *Gadd45a*, and *Prdx4*, among others. These genes revealed significant alterations in several GO-BP and KEGG pathways implicated in inflammation, cellular stress, synaptic plasticity, emotionality, and regeneration. Additionally, we found that incubation of primary neuronal cultures in DDW-containing buffer ameliorated Ca^2+^ influx and mitochondrial potential in a toxicity model, suggesting the involvement of mitochondrial mechanisms in the effects of decreased deuterium levels. Thus, aging induced profound brain molecular changes that may at least in part contribute to LLD pathophysiology. Reduced deuterium intake exerted modest but significant effects on LLD-related behaviors in aged mice, which can be attributed to, but not limited by ameliorated mitochondrial function and changes in brain gene expression.

## 1. Introduction

Late-life depression (LLD) presents a significant social and economic burden, particularly as the global population continues to age. Affected individuals experience psychological distress and a reduced quality of life [1,2,3]. Depressed older adults incur substantially higher healthcare costs than their non-depressed peers [4,5]. Untreated LLD is correlated with cognitive decline, disability, and diminished social support [6,7]. Thus, there is a growing need for improved interventions to mitigate the extensive medical and social burden of LLD.

In comparison with depressive symptoms in other age populations, LLD is often characterized by specific clinical symptoms, such as agitation, cognitive impairments, chronicity, and is frequently accompanied by somatic comorbidities [8,9,10]. It is associated with the characteristics of LDD biochemical changes in the brain, such as increased sterile inflammation [11], decreased markers of synaptic plasticity, e.g., of brain-derived neurotrophic factor (BDNF), and elevated markers of oxidative stress [12]. In comparison to other forms of depressive disorder, LLD is linked to age-related neurodegenerative and vascular processes [13,14] and has a poorer response to antidepressants [7,9,12]; as such, it requires different treatment approaches.

As the neurobiological basis of LLD cannot be fully addressed using neuroimaging methods, post-mortem brain analysis, or by studying peripheral changes, the use of animal models is indispensable for understanding the molecular mechanisms of this disorder [15,16]. Despite robust human data, the paucity of mechanistic animal studies has limited their therapeutic translation. In addition, advancing LLD treatment requires experimental validation of the underexplored pathways and biomarkers in aged animal models. However, few animal studies have recapitulated the key features of LDD, including anhedonia and decreased ability to experience pleasure, which are core symptoms of major depressive disorder (MDD) [17], and investigated the molecular determinants of LLD in the brain. Moreover, animal studies have mostly validated human post-mortem findings on LLD, while existing gaps in the application of animal models limit the progress in drug research and development in this field.

Available mechanistic studies on LLD have addressed changes in the hippocampus that play a major role in the mechanisms of depressive disorders [18,19]. For example, comparisons between 3- and 5-month-old mice and aged 18-month-old mice revealed widespread transcriptional changes in the hippocampus that are primarily associated with immune function, synaptic plasticity, and intracellular signaling [19], although direct links to depressive phenotypes and hedonic deficit remain underexplored. Studies performed on the prefrontal cortex, another key brain structure involved in the mechanisms of depression [20,21,22], revealed significant age-related shifts in this brain structure.

Reported aging-related gene expression changes are likely to underlie increased neuroinflammation, mitochondrial dysfunction, and compromised neuroplasticity in the elderly. It was shown that genes encoding pro-inflammatory factors, such as *C1qa*, *C1qc*, *Cxcl10*, *Tlr2,* and *Il33,* increase their expression in the brain from midlife, reflecting neuroinflammatory processes [23,24,25,26]. Genes involved in mitochondrial stress responses, such as *Ucp2* and *Txnrd1*, and genes encoding factors of antioxidant defenses, e.g., *Sod2* and *Gpx1*, were shown to be over-expressed in old age. Aging was shown to alter expression of genes encoding the metabolic regulators *Rps6kb1*, *Prkaa1*, *Igf1*, and *Irs1* [24]. Conversely, expression of genes encoding neuronal and synaptic plasticity such as *Arc*, *Egr1*, *Fos*, *Syn1*, and *Dlg4* decline with age, potentially indicating compromised cognitive and synaptic function [26]. Markers of astrogliosis and microglial activation *Gfap*, *Serpina3n*, and *Aif1* are over-expressed in elderly [23], while gene expression of the myelin-related molecules *Mbp*, *Mag*, and *Plp1* show mixed trends, potentially reflecting brain demyelination in aging [25]. Gene expression of neurotrophine *Bdnf* was shown to be lowered in the brains of older adults [12].

The role of the environment is an important aspect of studies on aging and aging-related CNS morbidities. Environmental and geographical conditions may significantly influence the risk of developing LLD [27,28,29,30]. Increased depressive symptoms can be associated with air pollution [31], water quality [32], insolation levels [33,34,35], annual fluctuations in air temperature [36,37], and the content of certain minerals in soil and water [38,39,40]. Severe problems related to rapid climate change further emphasize the role of environmental factors in this context [41,42].

Some environmental hazards are not self-evident; thus, careful investigation of new potential natural factors contributing to health risks is of particular importance. Unexpectedly, one of them can be the isotope composition of drinking water, namely the level of the hydrogen isotope deuterium. Natural water is a mixture of nine water isotopologues formed by stable isotopes of hydrogen [^1^H, protium (H) and ^2^H, deuterium (D)] and oxygen (^16^O, ^17^O, and ^18^O). Deuterium is the most abundant isotope of water [43,44] and can be found in a broad range of concentrations, from 90 to 155 ppm [43]. The abundance of water isotopologues in environmental water, expressed as the deviation (δ) relative to the international standard ‘Vienna Standard Mean Ocean Water 2′ (VSMOW2), varies by location and climatic conditions owing to isotopic fractionation during the evaporation–condensation process as air masses move inland over topographic features [44,45,46]. Remarkably, epidemiological and pre-clinical studies have revealed a correlation between deuterium consumption and the development of depressive syndrome [47].

Deuterium content decreases with distance from the ocean and altitude and is influenced by latitude, humidity, and seasonal temperature [48,49,50]. This variation is reflected in tap water, as shown in studies conducted across the United States [48]. A recent United States study linked higher deuterium levels in drinking water to increased adult depression rates, especially in coastal areas [47]. The lowest deuterium levels are found in Antarctica, where the Standard Light Antarctic Precipitation 2 (SLAP2) contains 43% less deuterium than VSMOW2 [46]. Even minor isotopic variations can influence mitochondrial function by affecting proton-coupled electron transport, ROS production, and ATP synthesis [51,52,53,54,55,56].

Deuterium-depleted water (DDW, 90–100 ppm) stabilizes oxidative metabolism and reduces mitochondrial stress, whereas elevated deuterium levels can be harmful [57,58,59]. Differentiated PC12 cells treated with 50–100 ppm DDW prior to hydrogen peroxide exposure showed improved viability, reduced apoptosis, and enhanced antioxidant defenses [60], which was further confirmed in cancer cell lines [61,62,63]. Systemic in vivo studies have revealed the sound anti-diabetic effects of DDW (125 ppm) in rats [64] and DDW (104 ppm) in humans [65].

Deuterium most profoundly affects basic cellular processes, such as membrane fluidity and receptor dynamics, influencing neurotransmitter release and synaptic transmission [52,53]. Increased membrane viscosity may slow neurotransmitter turnover, contributing to MDD [66]. In mice, DDW with the lowest deuterium content available in nature (~90 ppm) reduced behavioral despair and anhedonia and also normalized key factors of MDD development [67,68], improving REM sleep, increasing hippocampal neurogenesis, and SERT expression [47].

Given that mitochondrial dysfunction, common in LLD [69], is associated with oxidative stress, neuronal decline and gene expression of markers of aging [70,71,72,73,74,75,76,77,78], we hypothesized that changes in deuterium content in drinking water can alter susceptibility to LLD-like manifestations. Therefore, first, using a previously established mouse model of senile depression [79,80,81], we performed Illumina gene expression profiling in the hippocampi and prefrontal cortexes of 18-month-old C57BL/6J mice compared to 3-month-old controls. Previous studies have shown that 18-month-old C57BL6J male mice exhibit LLD-like key behaviors, such as anhedonia, helplessness, reduced exploratory motivation, and increased anxiety [79,82]. Next, the 18-month-old C57BL/6J group and a cohort of 12-month-old mice were allowed to consume deuterium-depleted water (DDW, ~90 ppm) or control water (CW, ~140 ppm) for 21 days.

The choice of deuterium content in DDW was based on the lowest concentration of deuterium available in nature, previously reported outcomes from chronic stress depression studies, and cell culture experiments [47,55,60,83]. Anhedonia was assessed using a sucrose preference test, helpless behavior was evaluated with the swim test, novelty exploration was studied with the novel cage test, and anxiety-like behavior was assessed in an elevated O-maze. Then mice were studied for LLD-like behaviors and Illumina gene expression in the hippocampus and prefrontal cortex. To confirm the Illumina results, qPCR assay was additionally applied to study expression of selected genes whose functional links with MDD and brain aging have been suggested by the literature.

## 2. Results

### 2.1. Deuterium and Mineral Analysis of the Samples

Deuterium and mineral measurements are detailed in the Supplementary file, Tables S1 and S2. These data show similar mineral content of CW and DWD. The biological analysis of the samples indicated an absence of contamination in the CW and DDW utilized in the study (see Supplementary File, Table S3).

### 2.2. A Comparison of Brain Gene Expression Between 18-Month-Old and 3-Month-Old Mice

**Figure 1:**
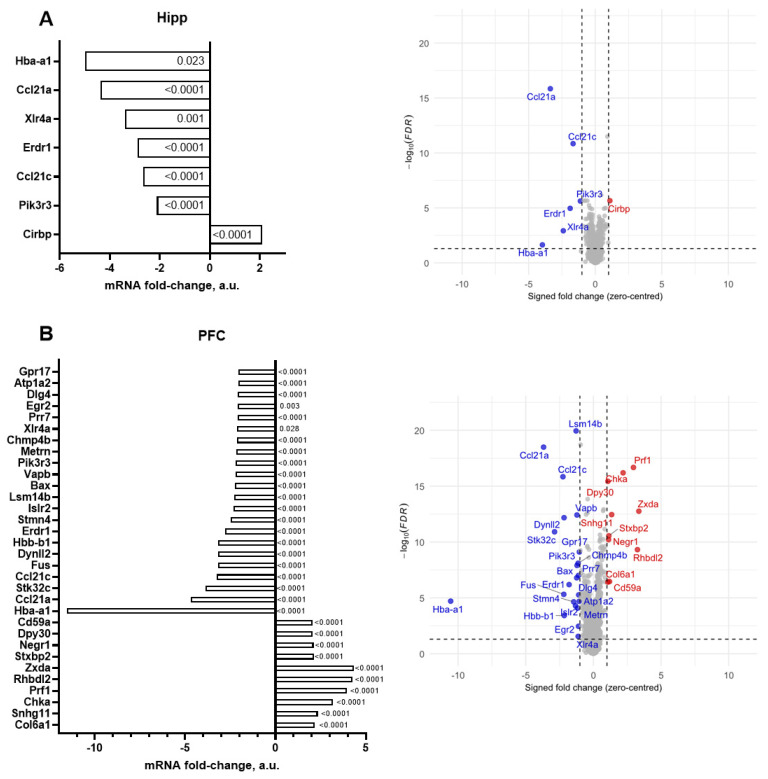
Illumina gene expression profiling of the brains of old and young mice. For the (**A**) hippocampus and (**B**) prefrontal cortex, genes with significantly altered mRNA expression, |fold change| ≥2, are shown in the aged group of mice in comparison to the young mice (see the ms text). Step-up FDR-corrected *p*-values (DDW vs. CW) are shown. Red indicates significantly upregulated genes, blue indicates significantly downregulated genes with FDR-corrected *p* < 0.05 (horizontal dashed line). All *n* = 5.

Using a criterion of ≥1.25-fold change, we found that 323 genes were significantly altered in the hippocampus, and 624 were significantly altered in the prefrontal cortex (*p* < 0.05, FDR-corrected). Using a criterion of ≥2.0-fold change, gene expression profiling of the hippocampus identified significant alterations in the expression of seven genes, with six genes exhibiting upregulation and one gene demonstrating downregulation by more than 2-fold compared to the control values (*p* < 0.05, FDR-corrected; Figure 1A, Table 1). Using the same criterion, 32 genes exhibited significant changes in expression, with 10 genes significantly upregulated and 22 genes significantly downregulated in the prefrontal cortex (*p* < 0.05, FDR-corrected; Figure 1B, Table 1). The outcome from analysis based on various combinations of fold changes from ≥1.25 to ≥2.5 and FDRs of <0.001, <0.01, and <0.05 are presented in the Supplementary file, Table S4.

For the ≥2.5-fold threshold in expression changes, 5 genes were significantly downregulated in the hippocampus and 13 genes showed significantly altered expression in the prefrontal cortex (Table 1).

Remarkably, for the ≥2.0-fold change, 1 out of 7 genes whose expression was upregulated was revealed by the Illumina assay in the hippocampus, and 10 upregulated genes out of 32 genes were found in the prefrontal cortex.

There were overlapping changes in significantly altered gene expression (fold change ≥ 2.0) between the hippocampus and prefrontal cortex. Five genes were found to be significantly altered in both brain regions (Table 2 and Table 3).

A comparison of Table 2 and Table 3 shows that the majority of significantly altered genes with ≥2.0 changes could be found in the prefrontal cortex, and five out of seven genes overlapped with the changes found in both brain structures.

### 2.3. Effects of DDW Exposure on Parameters of Emotionality

**Figure 2:**
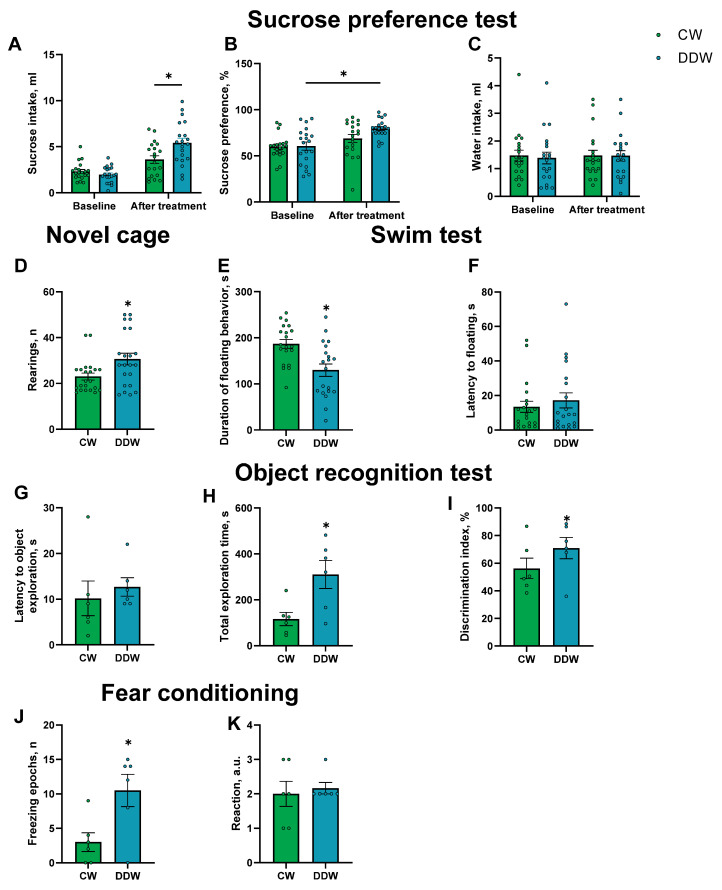
DDW-exposed mice display increased hedonic sensitivity, novelty exploration, and diminished helpless behavior. (**A**) The sucrose intake of DDW mice was significantly higher than that of CW mice after 21 days of housing on DDW. (**B**) No group differences in water intake were found in this assay. (**C**) Sucrose preference was significantly elevated in the DDW group following a 21-day exposure to DDW. In the sucrose preference test, no baseline differences in any measured parameters were detected between the groups (**A**–**C**). (**D**) DDW mice exhibited significantly more rearing in the novel cage test than CW animals, suggesting increased novelty exploration in the former group. On day 2 of the swim test, (**E**) a significantly shorter duration of floating behavior was noted in the DDW group compared to CW mice, and (**F**) no group differences were found in the latency to the first floating episode. (**G**) No group differences were shown in the latency to explore object. (**H**) Total exploration time was significantly longer in the DDW group compared to CW mice. (**I**) No group differences were found in the object discrimination index, which was significantly higher in the DDW group compared to chance level. (**J**) Percent of time spent freezing was significantly higher in the DDW group compared to CW mice. (**K**) No group differences were found in reaction to foot shock. * *p* < 0.05, repeated measures two-way ANOVA with post hoc Šídák’s test, or Mann–Whitney test, or unpaired *t*-test; *p* < 0.05, one-sample *t*-test. CW—control water, DDW—deuterium-depleted water, a.u.—arbitrary units. All *n* = 20 for 18 m.o. mice and all *n* = 6 for 12 m.o. mice. Bars are mean ± SEM.

In the sucrose preference test, two-way ANOVA demonstrated a significant interaction between time (day 0 vs. day 21) and housing on the effect of DDW (F1,38 = 7.61, *p* = 0.009, repeated measures two-way ANOVA, Figure 2A). Post hoc Šídák’s comparisons indicated that baseline sucrose intake did not differ between the groups under baseline conditions (*p* = 0.7420, Šídák’s test). Following housing in DDW, sucrose intake in the DDW group was significantly higher than that in the CW group (*p* = 0.0013, Šídák’s test). The analysis revealed a significant effect of time on sucrose preference (F1,38 = 16.59, *p* = 0.0002, repeated measures two-way ANOVA; see Figure 2B). Neither housing on DDW (F1,38 = 2.33, *p* = 0.14, repeated measures two-way ANOVA) nor the interaction between time and housing on DDW (F1,38 = 2.16, *p* = 0.15, repeated measures two-way ANOVA) exhibited a significant effect. Post hoc analysis showed a significant increase in sucrose preference following housing on DDW in the corresponding group (*p* = 0.0007, Šídák’s test), whereas no significant change was observed in CW mice (*p* = 0.14, Šídák’s test). The baseline values were comparable between the groups (*p* > 0.9999, Šídák’s test). No group differences were observed in water intake (time: F1,38 = 0.04, *p* = 0.84; housing on DDW: F1,38 = 0.06, *p* = 0.80; interaction: F1,38 = 0.04, *p* = 0.84, repeated measures two-way ANOVA; Figure 2C), suggesting that general liquid intake did not affect the outcome of this assay.

Two-way ANOVA revealed a significant interaction between time and housing on DDW in liquid intake (F1,38 = 5.96, *p* = 0.02, repeated measures two-way ANOVA). Post hoc analysis confirmed a significant increase in liquid consumption in the DDW group after housing on DDW compared to baseline consumption (*p* < 0.0001, Šídák’s test). These findings suggest that the observed changes in sucrose preference were not attributable to alterations in overall fluid consumption and that the increase in sucrose consumption was specifically induced by DDW exposure.

In the novel cage test, the total number of rearings in DDW mice was significantly greater than that observed in the CW group (U = 148, *p* = 0.027, Mann–Whitney test, Figure 2D), suggesting an enhancement in exploratory behavior following housing on DDW. Rearing counts were evaluated across one-minute intervals. No significant interaction effect was detected (F_4,168_ = 0.81, *p* = 0.52, repeated measures two-way ANOVA; Supplementary Figure S1). Significant main effects of time, housing on DDW, and animal were found on this parameter (time: F_3.68,154.4_ = 13.04, *p* < 0.0001; DDW: F_1,42_ = 6.60, *p* = 0.014; animal: F_42.168_ = 4.05, *p* < 0.0001, repeated measures two-way ANOVA). Post hoc Šídák’s multiple comparisons test showed no statistically significant differences at any individual time point in the number of rearing behaviors observed in the novel cage test (all *p* > 0.05, Šídák’s test). However, there was a trend suggesting increased rearing in the DDW group compared to that in the CW group during the first minute (*p* = 0.084, Šídák’s test).

In the O-maze, no significant group differences were observed in the time spent in the open arms (U = 243, *p* = 0.3515, Mann–Whitney test, Supplementary Figure S2A) or the latency to exit the open arms (U = 170.5, *p* = 0.4224, Mann–Whitney test, Figure 2F and Figure S2B) or the number of exits in the open arms (U = 254, *p* = 0.4843, Mann–Whitney test, Supplementary Figure S2C). Significant main effects of time, housing on DDW, and animal were identified for this parameter, suggesting no changes in anxiety-like behavior in DDW-exposed mice in our study.

Behavioral helplessness was assessed using the swim test. On the first day, the latency to the initial floating episode did not exhibit a significant difference between the groups (U = 137.5, *p* = 0.0923, Mann–Whitney test, Supplementary Figure S2D). Similarly, no significant differences were observed between the groups in terms of the duration of floating behavior (*p* = 0.1820, unpaired *t*-test, Supplementary Figure S2E). A significant reduction in the duration of floating was noted in the DDW group compared to in the CW group on the second day of the test (*p* = 0.0014, unpaired *t*-test, Figure 2E), indicating diminished helpless behavior in mice housed in the DDW condition. However, the latency to floating did not differ significantly between the groups (U = 196, *p* = 0.92, Mann–Whitney test, Figure 2F).

In the object recognition test conducted on 12 m.o. mice, no significant differences were found in the latency to explore objects (*p* = 0.573, unpaired *t*-test; Figure 2G) and in the object discrimination index (*p* = 0.201, unpaired *t*-test; Figure 2I). However, the DDW group but not the CW group had a significantly higher discrimination index when compared to chance level (*p* = 0.043 and *p* = 0.438, one sample *t*-test; Figure 2I). Mice housed with DDW showed significantly longer total durations of exploration compared to the CW group (*p* = 0.0165, unpaired *t*-test; Figure 2H).

On day 2 of the fear conditioning test, a significantly higher percentage of time spent freezing was observed in the 12 m.o. DDW group compared to the CW mice (*p* = 0.0198, unpaired *t*-test; Figure 2J). No significant differences were revealed in reaction to foot shock in arbitrary units (U = 16, *p* > 0.999, Mann–Whitney test, Figure 2K).

No significant group differences were found in parameters of anxiety-like behavior in the dark–light box (Supplementary Figure S3).

### 2.4. Illumina Gene Expression Profiling of Old Mice Exposed to DDW or CW

**Figure 3:**
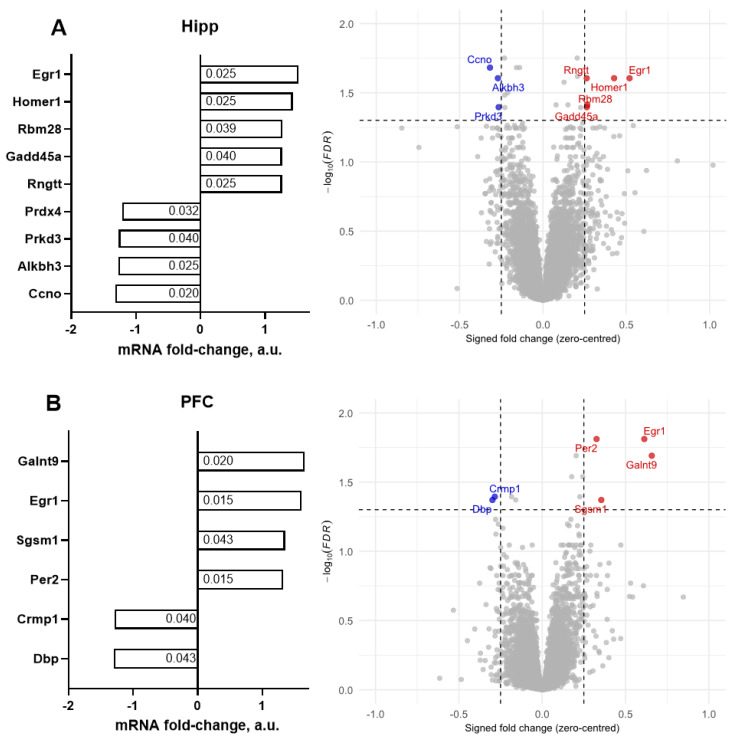
Illumina gene expression profiling of the brain of old mice exposed to DDW or CW. For the (**A**) hippocampus and (**B**) prefrontal cortex, genes with significantly altered mRNA expression |fold-change| ≥ 1.25 are shown in the DDW group in comparison to CW mice (see the ms text). Step-up FDR-corrected *p*-values (DDW vs CW) are shown. Red—significantly upregulated genes, blue—significantly downregulated genes, with FDR-corrected *p* < 0.05 (horizontal dashed line). All *n* = 5.

The total number of genes whose expression was significantly altered in the hippocampus was 21 and 14 in the prefrontal cortex, 35 in total (*p* < 0.05, FDR-corrected). Using a criterion of ≥1.25-fold change, gene expression analysis of the hippocampus identified significant alterations in the expression of nine genes, with five upregulated and four downregulated genes (*p* < 0.05, FDR corrected; Figure 3A, Table 4). The outcome from analysis based on various combinations of fold changes from ≥1.25 to ≥2.5 and FDRs of <0.001, <0.01, and <0.05 is presented in the Supplementary file, Table S4.

Accordingly, in the prefrontal cortex, six genes exhibited significant expression changes, with four genes significantly upregulated and two genes significantly downregulated (*p* < 0.05, FDR-corrected; Figure 3B and Table 4).

### 2.5. Pathway Analysis and Functional Roles of Differentially Expressed Genes

Functional roles of differentially expressed genes are summarized in Table 5 and Table 6. In addition, the pathway analysis revealed their involvement in key cellular processes. In the hippocampi of old mice compared to young mice, GSEA analysis revealed no significantly altered GO-BP pathway enrichment and two significantly altered KEGG pathways. Although no GO-BP pathways were significant after FDR correction, strong trends were observed for “Erythrocyte development” (*p* = 0.081, normalized enrichment score (NES) = −2.28), “Positive regulation of cell migration” (*p* = 0.085, NES = −1.81), “Antigen processing and presentation of exogenous peptide antigen” (*p* = 0.081, NES = 1.99), and “Complement activation, classical pathway” (*p* = 0.091, NES = 1.95). Mirroring these findings, KEGG pathway analysis highlighted significantly downregulated “Malaria” (*p* = 0.015, NES = −2.25) and “African trypanosomiasis” (*p* = 0.018, NES = −2.27) gene sets in old mice, which are primarily driven by complement components.

In the prefrontal cortexes of old mice, one GO-BP pathway and four KEGG pathways were found to be significantly altered. The “DNA repair” GO-BP pathway (*p* = 0.018, NES = 1.90), and KEGG “Allograft rejection” (*p* = 0.047, NES = 1.99) and “Graft-versus-host disease” (*p* = 0.047, NES = 2.02) pathways, were significantly upregulated. As in the hippocampus, “Malaria” (*p* = 0.0019, NES = −2.46) and “African trypanosomiasis” (*p* = 0.047, NES = −2.30) KEGG pathways were significantly downregulated. Together, these findings reflect age-related neuroinflammation regulation and a shift towards chronic immune surveillance.

In the hippocampi of the DDW group, 8 GO-BP terms and 25 KEGG pathways were significantly altered (see Table 7 and Table 8). Among them, the following synaptic plasticity programs were upregulated: localization of neurotransmitter receptors (*p* = 0.001, NES = 2.24), GABAergic signaling (*p* = 0.02, NES = 2.03), long-term memory genes (*p* = 0.029, NES = 1.99), and dendrite development (*p* = 0.029, NES = 2.03). KEGG echoes this with actin cytoskeleton regulation (*p* = 0.004, NES = 1.76), cAMP/cGMP signaling (*p* = 0.005, NES = 1.70, and *p* = 0.005, NES = 1.75, respectively), and oxytocin signaling (*p* = 0.004, NES = 1.83), which are canonical pathways of structural and functional plasticity.

Two GO-BP and three KEGG pathways were significantly altered in the prefrontal cortex in the DDW-exposed group of aged mice. The “Adult walking behavior” GO-BP pathway was significantly downregulated (*p* = 0.045, NES = −2.02) and “Chaperone-mediated protein folding” was significantly upregulated (*p* = 0.045, NES = 0.06). KEGG pathways “AGE-RAGE signaling pathway in diabetic complications” (*p* = 0.006, NES = 1.93), “FoxO signaling pathway” (*p* = 0.007, NES = 1.84), and “Human T-cell leukemia virus 1 infection” (*p* = 0.044, NES = 1.58) were significantly upregulated in the DDW group, suggesting upregulation of stress response and survival signals.

### 2.6. qRT-PCR Gene Expression Profiling of Selected Genes

**Figure 4:**
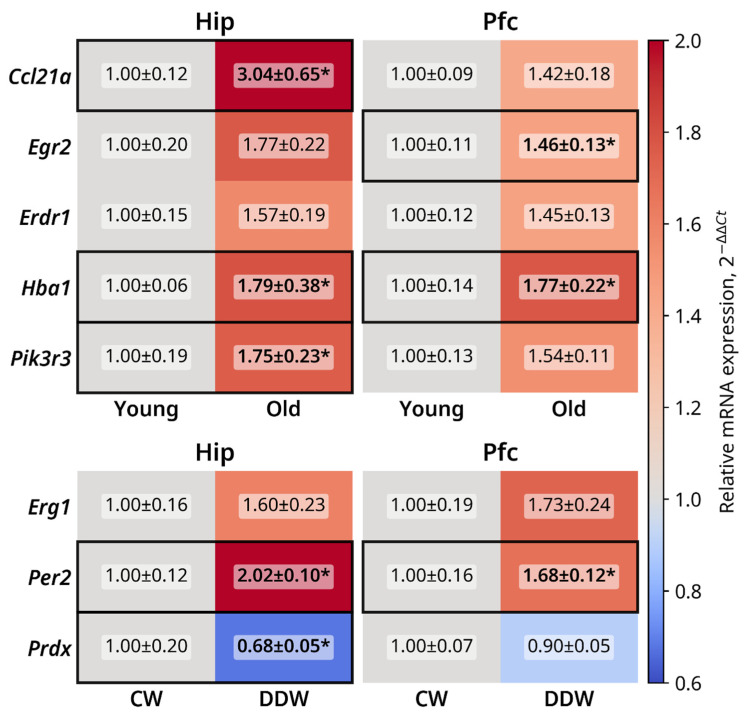
Heatmap of qRT-PCR expression of differentially expressed genes selected from the Illumina datasets. Old mice showed significantly higher mRNA levels of *Ccl21a* and *Pik3r3* in the hippocampus and *Egr2* in the prefrontal cortex, as well as significantly increased *Hba-a1* levels in both structures. Expression of *Per2* in the hippocampus and the prefrontal cortex was significantly increased in the DDW-treated group, as well as expression of *Prdx4* in the hippocampus. * *p* < 0.05, Mann–Whitney test or unpaired *t*-test; CW—control water; DDW—deuterium-depleted water; Hip—hippocampus; Pfc—prefrontal cortex. All *n* = 5, data are shown as mean ± SEM.

qRT-PCR assay was performed on a subset of genes that showed the most prominent differential expression in the Illumina dataset or/and that were changed in their expression both in the hippocampus and prefrontal cortex. Heatmaps of gene expression changes are shown in Figure 4 and data plots are presented in Supplementary Figure S4.

A significant increase was observed in hippocampal *Ccl21a* expression (*p* = 0.02, Mann–Whitney test) in Figure 4 in the old mice compared to the young mice, as well as a strong trend for increased expression in the prefrontal cortex (*p* = 0.095, Mann–Whitney test). *Erg2* expression was significantly elevated in the prefrontal cortexes of aged mice (*p* = 0.03, Mann–Whitney test), and an increase in its expression in the hippocampus was observed (*p* = 0.056, Mann–Whitney test). Trends for increased *Erdr1* expression in the prefrontal cortex and hippocampus were also observed (*p* = 0.056 and *p* = 0.095, respectively, Mann–Whitney U test). The expression of *Hba1* was significantly higher in the hippocampus and prefrontal cortex (both *p* = 0.016, Mann–Whitney test) in old mice than in young mice. Finally, *Pik3r3* expression was significantly elevated in the hippocampi of the old group (*p* = 0.03, Mann–Whitney test), with the same strong trend observed in the prefrontal cortex (*p* = 0.056, Mann–Whitney test).

Strong trends for elevation of *Egr1* expression in the hippocampus and prefrontal cortex were revealed in the DDW group (both *p* = 0.056, Mann–Whitney test). Expression of *Per2* was significantly higher in both the hippocampi and prefrontal cortexes (both *p* < 0.01, Mann–Whitney test) of old mice housed with DDW compared to the CW group. *Prdx4* expression in the hippocampus was significantly lower in the DDW group than in the CW group (*p* = 0.03, Mann–Whitney test); no significant group differences were found for its expression in the prefrontal cortex (*p* = 0.42, Mann–Whitney test).

### 2.7. Protective Effects of Decreased Deuterium Levels on the Protonophore FCCP-Induced Ca^2+^ Influx in a Model of Neurotoxicity and Neuronal Damage in a Rat Neuronal Culture

**Figure 5:**
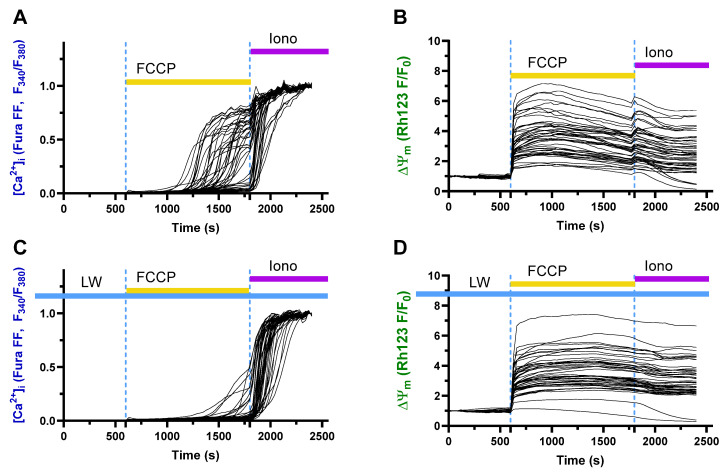
Protective effects of decreased deuterium levels on FCCP-induced Ca^2+^ influx and mitochondrial potential in neuronal primary culture (representative curves). Following FCCP protonophore treatment, (**A**) there was a sharp increase in intracellular Ca^2+^ concentration and (**B**) a decrease in mitochondrial membrane potential in a primary neuronal culture that was maintained on a regular buffer. (**C**) Cell culture pre-incubated in DDW-containing buffer had a significantly blunted increase in intracellular Ca^2+^ concentration, and (**D**) partly preserved the drop in membrane potential.

**Figure 6:**
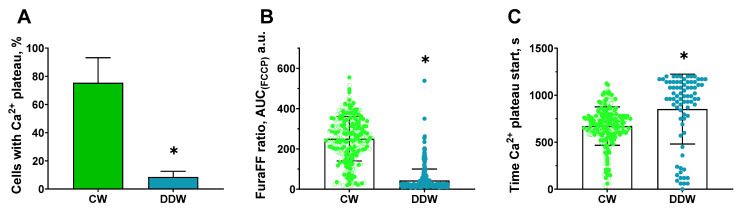
The number of cells reaching the calcium plateau and FuraFF fluorescence in DOW-incubated primary neuronal cultures treated with FCCP. In comparison with cell culture maintained on a regular buffer, cell culture incubated in a buffer with a decreased deuterium level displayed (**A**) a significantly decreased number of cells reaching the calcium plateau, (**B**) a significantly decreased AUC of FuraFF fluorescence level (CW-based buffer: *n* = 931, DDW-based buffer: *n* = 919), and (**C**) a significantly increased time to reach the calcium plateau (CW-based buffer: *n* = 694, DDW-based buffer: *n* = 85). * *p* < 0.001, CW- vs. DDW-based buffer. Statistical tests: (**A**) * *p* < 0.001, Mann–Whitney U test applied to experiment-level counts; (**B**,**C**) * *p* < 0.0001, robust LMM. DDW-cultured cells were maintained in a buffer containing deuterium-depleted water. Data are from four independent experiments (each comprising one control and one DDW dish; see ms text).

Treatment with FCCP induced a rapid increase in intracellular Ca^2+^ levels (Figure 5A) and a decrease in mitochondrial membrane potential (Figure 5B). These changes were ameliorated in the culture pre-incubated in DDW-containing buffer (Figure 5C,D). In addition, incubation in DDW buffer significantly decreased the number of cells that reached the calcium plateau as shown by the analysis of four assays merged together (Figure 6A, *p* < 0.01, Mann–Whitney test). Robust linear mixed-effects (LMM) modeling with condition as a fixed effect and experiment as a random intercept showed that the DDW group had a significantly decreased AUC of FuraFF fluorescence compared to the CW cultures (Figure 6B, *p* < 0.0001, robust LMM). The same model applied to the time elapsed to reach the start of the calcium plateau, which indicated a stronger increase in the DDW-treated cell culture than in the control culture (Figure 6C, *p* < 0.0001, robust LMM). For AUC analysis, the following numbers of cells were evaluated in experiments 1–4. CW-based buffer: 1st assay, *n* = 257, 2nd assay, *n* = 258, 3rd assay, *n* = 261, and 4th assay, *n* = 155 (total: *n* = 931); DDW-based buffer: 1st assay, *n* = 202, 2nd assay, *n* = 242, 3rd assay, *n* = 307, and 4th assay, *n* = 168 (total: *n* = 919). For the time to reach the calcium plateau, cell counts in experiments 1–4 were, for the CW-based buffer, 1st assay, *n* = 249, 2nd assay, *n* = 158, 3rd assay, *n* = 155, 4th assay, *n* = 132 (total: *n* = 634); DDW: 1st assay *n* = 26, 2nd assay, *n* = 41, 3rd assay, *n* = 35, 4th assay, *n* = 10 (total: *n* = 85).

## 3. Discussion

In this study, 18-month-old male mice utilized as a model for LLD exhibited significant downregulation of over 398 genes in the hippocampus and prefrontal cortex, alongside significant upregulation of over 476 genes in these brain regions, compared to 3-month-old mice. This was determined using a criterion of 1.25-fold change, which may correspond to minimal physiological relevance of alerted gene expression. When a criterion of greater than 2.0-fold change was applied, it was observed that downregulation, rather than upregulation, was predominant in the gene expression changes, primarily occurring in the prefrontal cortex of old mice. Nearly all genes altered in the hippocampus were also found to be altered in the prefrontal cortex. In another study, 18-month-old male mice consumed drinking water containing the lowest naturally occurring concentration of deuterium. This intervention had a subtle but significant effect on behavioral manifestations of LLD-like changes, improving hedonic deficits, novelty exploration, and helplessness, while anxiety-like behavior was unchanged. Illumina gene expression profiling revealed significant alterations in the expression of 35 genes in total and of 15 genes when a 1.25-fold change was used. The majority of genes were altered in the hippocampus but not in the prefrontal cortex. In both Illumina assays, the identified genes were associated with synaptic plasticity, circadian regulation, depression, stress, and antioxidant factors. The pathway analysis suggested that aging increased complement activation and MHC-II antigen presentation and dampened erythroid, migratory, and synaptic-plasticity routes. DDW-exposed aged mice showed pathway changes associated with increased dendritic remodeling and involvement of GABAergic, actin-cytoskeleton, and immune-surveillance pathways.

These and other molecular changes may contribute to the observed modifications in emotionality revealed in the two studies. Although it is difficult to hypothesize a direct relationship among a limited number of identified differentially expressed genes with the observed behavioral changes, it is remarkable that significant molecular and behavioral alterations can be exerted by a combination of these subtle natural factors, such as aging and intake of drinking water with the lowest possible natural concentrations of deuterium. Clearly, the behavioral changes reported here can be underpinned not only by altered gene expression but also by regulatory mechanisms at the protein level, as well as epigenetic and post-translational modifications. As the Ca^2+^-influx experiment on a neuronal cell culture has shown, a decrease in cellular deuterium level alters mitochondrial processes that are likely to contribute to the behavioral changes observed here as well.

Our study with DDW revealed only a small number of differentially expressed genes. Small but significant changes in the expression of genes encoding proteins regulating membrane-associated processes, energy/carbon metabolism, and immune response pathways demonstrated the strongest correspondence to minor mRNA variations, in contrast to other proteins [176]. This may explain why the present study, along with numerous studies in the literature, has shown that fold changes of approximately 1.25 in gene expression can be biologically meaningful [177,178,179]. Our previous Illumina assays revealed large proportions of significantly altered genes whose expression was changed at 1.25–1.5-fold in animal models of stress and was accompanied by profound behavioral and physiological abnormalities [180,181]. Recent evidence of more robust and reproducible physiological processes accompanying subtle versus pronounced changes in gene expression was considered to better reflect the network connectivity of altered genes [178,182]. It should be noted that the present study did not involve major challenges but used naïve, albeit aged, animals and environmental exposure to water with natural characteristics, allowing the anticipation of modest changes in genes and behavior in experimental mice.

Previous studies have reported age-related gene expression changes in the brain. For example, Li et al. (2021) demonstrated altered expression of *Fgf2*, which is involved in neurogenesis and stress resilience, and *Cbln1*, a contributor to synaptic connectivity [31]. Here, both genes showed reduced expression in the hippocampi of aged mice, potentially implicating them in the LLD-like behaviors described here. In the present study, aged mice had a downregulated *Hba-a1* gene, which encodes the hemoglobin subunit alpha and is implicated in neurovascular coupling and mitochondrial respiration, whose compromised functions can lead to impaired brain energy metabolism, a factor of MDD [183]. Old mice showed increased expression of *Cirbp* (cold-inducible RNA-binding protein) which regulates circadian rhythm and inflammatory responses stimulating IL-6 production via the NF-κB pathway [184], thus increasing neuroinflammation, a hallmark of MDD.

Aged mice revealed decreased expression of chemokines *Ccl21a* and *Ccl21c*, whose dysregulation could alter microglial activation, upregulate interferon-α/β, and suppress NK cell activity, which is consistent with findings of aberrant immune signaling in MDD [185]. *Erdr1* (erythroid differentiation regulator 1) is an anti-inflammatory gene with an apoptotic regulatory function; its dysregulation, shown here in aged animals, may tip the balance between neuroprotection and cell death [186]. We also showed diminished expression of *Xlr4a*, which is involved in chromatin remodeling and stress-induced epigenetic alterations in MDD, and of *Pik3r3*, which is directly involved in the PI3K-Akt signaling pathway. Compromised PI3K-Akt signaling affects synaptic plasticity and neuronal survival and is associated with MDD [187]. While it is difficult to conclude which of these changes reflect adaptation to destructive aging-related changes in the brain and which of them manifest these changes, the synthesis of gene function and pathway interaction suggests alterations in immunity, mitochondrial regulation, neuroplasticity, and mechanisms regulating oxidative stress to be associated with an LLD-like syndrome in the employed mouse model.

In the current study, it was demonstrated that 21-day exposure of 18-month-old male mice to DDW enhanced sensitivity to reward, stimulated novelty exploration, and reduced behavioral helplessness, indicating an antidepressant-like effect exerted in senile animals [188]. Previous studies have established that mice of this age can serve as a model for LLD, displaying diminished sucrose preference, reduced exploration of a novel environment, increased immobility in the swim test, and increased anxiety-like behaviors [79,82]. Notably, many of these behavioral alterations were reversed following chronic administration of compounds with antidepressant properties [47,82]. The present study identified analogous effects of housing on DDW, with the exception of anxiety-like behaviors, which remained unchanged. An additional study in 12-month-old mice revealed ameliorated effects of DDW on contextual fear conditioning memory, a form of hippocampus-dependent learning, and improved object recognition, both of which decline with aging [189]. Remarkably, this study showed increased novelty exploration and unaltered anxiety-like behavior in the dark light box in DDW-exposed old animals, consistent with the findings reported in 18-month-old mice. Increased novelty exploration is considered an opposing behavior to depressive-like behavioral changes in laboratory mice [79,190]. These results align with previous experiments, indicating that decreasing the deuterium content in drinking water to the levels used here can reduce the behavioral, physiological, molecular, and histological hallmarks of stress-induced MDD-like syndrome in young mice [47].

Significant changes in gene expression in the limbic system found in our study suggest that DDW may modulate the molecular pathways involved in LLD [77,78]. We found that DDW-exposed old mice displayed elevated expression of *Egr1*, the downregulation of which is related to MDD and stress [148,149,150,179], as well as cognitive deficits [191,192]. DDW-exposed old mice revealed hippocampal upregulation of *Homer1a*, which regulates postsynaptic scaffolding and mitochondrial protection, which are related to the mechanisms of synaptic plasticity, neuroprotection, and MDD [151,152,153,193,194,195,196]. DDW-exposed mice also showed increased hippocampal expression of the *Gadd45a* gene, which supports neuronal survival and plasticity, and its deficiency causes neurodegenerative-like changes [155,197].

Gene expression of *Prdx4*, encoding an antioxidant factor, was decreased in the brain of the DDW-treated group, which may hypothetically reflect reduced oxidative stress in these mice [158]. DDW-exposed animals showed upregulation of *Galnt9* in the prefrontal cortex; notably, this gene is implicated in protective mechanisms against mitochondrial dysfunction [166]. In the prefrontal cortexes of DDW-exposed mice, we found a significant upregulation of the *Per2* gene, which regulates sleep and mood and is implicated in the mechanisms of MDD [170,171,198,199,200]. Another gene linked to anxiety/depression whose expression was significantly altered in the prefrontal cortexes of DDW-exposed old mice was *Crmp1* [173]. Notably, the qRT-PCR data were consistent with the findings obtained using the Illumina platform, thereby supporting the reliability of our transcriptomic results. Collectively, DDW exposure can modulate the expression of genes supporting synaptic plasticity, mitochondrial resilience, and circadian regulation, which leads to speculation about the role of these mechanisms in the antidepressant-like effects reported here.

To address the potential mitochondrial effects of DDW, we performed a primary cortical culture assay, which suggested that lowered deuterium levels in a buffer counteract protonophore-induced Ca^2+^ influx and a decrease in mitochondrial potential, indicating the antitoxic effects of DDW. These data suggest that mitochondrial mechanisms may underlie the protective effects of DDW, as reported in in vivo assays. The current findings contribute to the understanding of mechanisms, beyond alterations in brain gene expression, by which DDW influences mouse behavior. These results, although requiring follow-up measurements of potential changes in ΔΨm pH and ATP levels under the employed settings, are in line with the previously suggested role of D/H balance in mitochondrial functions [201,202,203]. Indeed, central insulin receptor-mediated processes and the previously demonstrated upregulation of GLUT4 under conditions of lowered deuterium levels [63,64,65] could underlie the mitochondrial effects of DDW and contribute to the behavioral effects of DDW in aged mice, as mitochondrial dysfunction is implicated in the neurobiology of LLD [204].

Despite the limitations of the study, such as the small sample size, use of microarrays, short duration of DDW exposure, lack of analysis of sex as a biological variable, correlational nature of some data, and the challenge of extrapolating modest transcriptomic changes to protein-level effects, we cite evidence showing that DDW exerts small mRNA changes which can have pathway-specific translational consequences in the brain and ameliorate behavior in a mouse model of LLD. The results from the Ca^2+^ influx cell culture assay suggest the role of mitochondrial regulation in the effects of DDW; however, this cannot be directly extrapolated through the in vivo context and requires an additional control of the pH and APP of the DDW buffer to rule out the contribution of pH changes in the observed effects. Finally, in our study, we did not observe any side effects of DDW at ppm 90. Indeed, studies on the negative effects of deuterium depletion in animals and humans is lacking; addressing this issue should be a priority in future experiments. In summary, as discussed above, the present study showed that relatively brief exposure to DDW produces subtle but significant changes in gene expression and behavior in a mouse model of LLD that would be of interest to investigate further as a potential environmental factor of this medical condition associated with aging.

## 4. Materials and Methods

### 4.1. Experimental Animals

The experiment was performed using 18-month-old male C57BL/6J mice, 12-month-old male C57BL/6J mice, and 3-month-old male C57BL/6J mice. The animals were from certified provider Charles River (Janvier, L’Arbresle Cedex, France). Mice were single housed and kept under a 12 h light–dark cycle (lights on: 21:00 h) with food and water ad libitum, under controlled laboratory conditions (22 ± 2 °C, 55% humidity). In order to minimize the possible influence of the environment, behavioral testing was performed during the dark period of the animal’s light cycle (after 9:00 h); other possible confounders were controlled as described elsewhere [205,206]. Specifically, a sufficient inter-test interval was set to avoid any potential interference between experimental manipulations and cage changes. Mice were allowed to adapt to the experimental room prior to behavioral evaluation for at least 1 h. Behavioral testing and killing of individual mice from different groups were carried out in an interchanging manner to avoid the influence of the day cycle and other biases. Animals were observed on each morning and evening of the experimental period. All experiments were carried out in accordance with the European Union’s Directive 2010/63/EU and Portuguese Law-Decrees, upon approval by Direccao Geral de Veterinaria, Ministerio da Agricultura, do Desenvolvimento Rural a das Pescas, license Nr. 685412, DG VGZ/VVP (S. 135), 0421/000/000/2013, and the Universidade de Lisboa on animal care and welfare (DGV-2009-10-22-00248216); they were compliant with ARRIVE guidelines (http://www.nc3rs.org.uk/arrive-guidelines, accessed on 22 September 2025). All efforts were undertaken to minimize potential suffering of the animals; the study did not have humane endpoints.

### 4.2. Study Flow

Following arrival from the supplier, five mice of the 3 m.o. and 18 m.o. subgroups were assigned to the first experiment with Illumina gene expression profiling and qRT-PCR assay. Additionally, 12 m.o. (*n* = 12) and 18 m.o. mice (*n* = 40) were randomized to the control water (CW, 140.7 ppm deuterium) and DDW (91.6 ppm deuterium) groups (both *n* = 6 for 12 m.o. mice and both *n* = 20 for 18 m.o. mice). DDW- and CW-housed 18 m.o. mice (both *n* = 5) were utilized for Illumina gene expression profiling and qRT-PCR validation. Mice in the DDW groups were allowed to consume deuterium-depleted water (DDW, ~90 ppm) or control water (CW, ~140 ppm) for 21 days, which was previously reported to exert physiological and molecular effects in a chronic stress paradigm [47]. In total, 62 mice were used; group size calculation was based on previous experiments with DDW [47].

Groups were randomized by body weight and coat score to ensure the absence of any a priori group differences. No criteria were set for including or excluding animals, as no mice were excluded from the analysis. Possible confounds were systematically controlled in both behavioral assays (see above) and molecular experiments. In the Illumina assays, RNA samples were distributed across different chips for proper randomization, and arrays were checked for similar gene intensity distributions to rule out potential outliers.

All mice were allowed an acclimatization period of 10 days before the Illumina study on naïve mice in the first experiment and prior to the second experiment, with DDW or CW exposure. CW and DDW had identical mineral content and no bacterial contamination, which was re-confirmed in the post-experimental assay of water samples (see Supplementary Tables S1–S3). In a preliminary experiment, a separate set of naïve mice was exposed to a 24 h free drinking paradigm using tap water, CW, or DDW to rule out possible differences in the consumption of either type of water; this revealed no such differences.

In the first study, 3- and 18-month-old mice (each group contained 5 mice) were sacrificed after an acclimatization period; their hippocampus and prefrontal cortex were isolated (see below) and frozen for subsequent RNA isolation and Illumina and qRT-PCR assays of selected genes (see below).

In the second experiment, a sucrose preference test was carried out to establish baseline sucrose preference in 18 m.o. mice on day 0. The mice were then housed for 21 days with CW or DDW under standard laboratory conditions. On day 21, the sucrose preference test was repeated after cessation of housing with CW/DDW and the sucrose preference test was repeated. Mice were then tested in a battery of behavioral tests: the novel cage test and elevated O-maze (day 22), and two sessions of the swim test (days 23–25). The experimenter was blind to the groups until the data analysis in all behavioral studies. On day 26, the mice were culled, and their brains were removed and dissected, as described elsewhere [188]. The prefrontal cortexes and hippocampi were isolated (see below) and kept frozen at −80 °C for subsequent RNA isolation and Illumina and qRT-PCR assays.

In the third experiment, 12 m.o. mice were housed for 21 days on CW or DDW under standard laboratory conditions. Mice were then tested in the dark–light box (day 22), the object recognition test (days 22–23), and fear conditioning (days 23–24) to evaluate the earlier phase of aging associated with LLD and cognitive impairment. The experimenter was blind to the groups until the data analysis in all behavioral studies.

### 4.3. Culling and Brain Dissection

Mice were euthanized through an overdose of a mixture comprising Ketanest (20%, Parke-Davis, Berlin, Germany) and Rompun (8%, BayerVital, Leverkusen, Germany), after a pre-exposure to CO_2_. Subsequently, the mice underwent transcardial perfusion with PBS, pH 7.4, followed by RNA later (Sigma Aldrich, St. Louis, MO, USA). The brains were excised and dissected, with the prefrontal cortexes and hippocampi isolated and preserved at −80 °C for subsequent RNA isolation and Illumina assay.

### 4.4. Illumina Gene Expression Profiling

In both studies, gene expression profiling was conducted using Illumina technology (Integra-Gen, Evry, France) on the hippocampus and prefrontal cortex of experimental groups of mice, as previously described [206]. Samples were randomly assigned to chips, ensuring that no two samples from the same group were placed on the same chip, thereby preventing confounding of experimental groups with chip effects [206]. The resulting microarray data underwent standard analytical procedures, including an assessment of overall array data quality and statistical evaluation of differentially expressed genes. The quality of the array data was confirmed, and the Gene Chip Operating System was employed to calculate signal intensities, detection calls, and their associated *p*-values for each transcription array. Gene expression was normalized to the expression of the housekeeping gene GAPDH, which showed consistent stability as validated in our laboratory using RefFinder [181] across multiple paradigms, including stress, neurodegeneration, and metabolic dysfunction [207,208,209,210,211]. Previous studies showed consistent outcomes from PCR assays in which GAPDH was used alone, or in combination with other reference genes [209]. Expression was calculated as fold changes relative to the CW mice. Illumina data were imported into Partek Genomics Suite and quantile normalized. Arrays identified as outliers through principal component analysis (PCA) were excluded from the dataset. Comparisons between experimental groups were performed using Partek Genomics Suite, with *p*-values adjusted for multiple testing using the step-up False Discovery Rate (FDR) method. In the study of a comparison old vs. young mice, the criteria for selecting differentially expressed genes were set as FDR *p* < 0.05 and |fold change| ≥ 2.0. In the study of a comparison CW- vs. DDW-exposed old mice, the criteria for selecting differentially expressed genes were set as FDR *p* < 0.05 and |fold change| ≥ 1.25. Each group in this study consisted of 5 animals. Additional analysis using various FDR thresholds and fold change rate were applied in each experiment.

### 4.5. Pathway Analysis

All analyses were performed in R 4.4.3 [212] under Bioconductor 3.21. Genes from the Illumina assay were ranked by a zero-centered signed linear fold change:

$$zFC=sign\left(FC\right)\left({\left|FC\right|}^{-1}\right)$$

Gene annotations were supplied by org.Mm.eg.db. To ensure a strictly unique order, each score was assigned an infinitesimal rank-proportional offset (ε = 10^−9^). Mouse annotations for the Gene Ontology—Biological Process (GO-BP) were obtained from the org.Mm.eg.db package of Bioconductor 3.21. For KEGG, Mus musculus pathway definitions were downloaded at runtime using KEGGREST 1.42 [213], following the REST interface of KEGG [214]. For both GO-BP and KEGG, terms smaller than 15 or larger than 500 genes were discarded to avoid unstable estimates.

Rank-based gene-set enrichment analysis (GSEA) was carried out with the fgseaSimple function from the fgsea 1.32.4 package [215]. For each comparison we executed, 3,000,000 label permutations were executed. The test returns a raw enrichment score (ES), which is normalized against its size-matched null distribution to yield a normalized enrichment score (NES), allowing comparison across pathways. For each library, the full vector of permutation *p*-values was adjusted using the Benjamini–Hochberg procedure [216]. Gene sets with FDR-adjusted *p* < 0.05 were regarded as significant.

### 4.6. Behavioral Tests

#### 4.6.1. Sucrose Preference Test

In this study, mice were provided with an eight-hour period during which they could freely choose between two bottles: one containing 1% sucrose solution and the other containing standard drinking water [205]. The bottles were weighed at both the commencement and conclusion of this period to calculate consumption. The test began at the onset of the animals’ dark (active) phase, specifically at 9:00. To mitigate any potential side-preference in drinking behavior, the positions of the bottles within the cage were alternated at the four-hour mark, halfway through the testing period. No prior deprivation of food or water was imposed before the test. To minimize liquid spillage during the sucrose test, the bottles were pre-filled and maintained in an inverted position for a minimum of 12 h prior to testing. Additionally, to equilibrate the air temperature between the room and the drinking bottles, the bottles were stored in the same room where the testing occurred. This precautionary measure was taken to prevent the physical effect of liquid leakage, which can result from increased air temperature and pressure inside the bottles when they are filled with liquids cooler than the ambient room air.

Percentage preference for sucrose was calculated using the following formula:

Sucrose Preference =100 × Volume(Sucrose solution)/(Volume(Sucrose solution) + Volume (Water))

#### 4.6.2. Novel Cage Test

The novel cage test was conducted to evaluate vertical exploration activity in a novel environment. A mouse was placed in a transparent plastic cage (14 × 21 × 27 cm) containing a small amount of fresh litter, under a light intensity of 5 lux. The number of rearing behaviors and the latency to the first rear were recorded over a 5 min period [205,211].

#### 4.6.3. The O-Maze

The O-maze apparatus (Technosmart, Rome, Italy) comprised a circular pathway with a runway width of 5.5 cm and a diameter of 46 cm, positioned 50 cm above the floor. Two opposing arms were enclosed by walls measuring 10 cm in height, and the illumination intensity was set at 25 lux. Anxiety-like behavior was evaluated using previously validated parameters as detailed in prior studies [217,218]. Mice were introduced into one of the closed arm compartments of the maze. The total duration spent in the open arms of the maze and the frequencies of visits to the open arms were recorded as established indicators of anxiety-like behavior during the initial 5 min period.

#### 4.6.4. Swim Test

Mice were subjected to two swimming sessions with a 24 h interval, as detailed in a previous study [188,218]. Each session lasted 6 min and involved placing a mouse in a transparent cylinder (Ø 17 cm) filled with water at 23 °C, with a water height of 13 cm and a cylinder height of 20 cm, under an illumination intensity of 25 lx. The duration of floating behavior was defined as the absence of any directed movements of the head or body, and this was assessed by an observer who was blinded to the identity of the animal. Both the latency to the first episode of floating and the duration of floating behavior were recorded.

#### 4.6.5. The Dark–Light Box

The apparatus consisted of a dark chamber and an illuminated chamber (600 lux). Mice were introduced to the dark compartment and were allowed to move freely between the two chambers. Latency to exit to the lit compartment, time spent therein and the number of exits to the lit box, as well as latency and number of risk assessment events, were recorded for 5 min as described elsewhere [205,217].

#### 4.6.6. Object Recognition Test

Mice were studied for new object recognition in a 2-day test, as described elsewhere [218]. The apparatus consisted of a plastic square arena (40 × 40 × 40 cm) with two objects, a “brush” and “flower”, 7 × 4 × 3 cm, placed symmetrically 2 cm away from the cage wall in the opposite corners of the arena. Lighting of 25 lx intensity was used. Objects used in the new object recognition test were either disposable and were new for each mouse (flowers were made from paper) or changeable and washable with water and mild detergent (plastic brush), so that any contamination (the smell of a preceding mouse) was virtually excluded. On day 1 of the test, two identical objects were used, and a mouse was placed in the arena at an equal distance from the objects and allowed to explore the area freely for 15 min. On day 2, one object was replaced by the new object and mouse was placed in the arena for 15 min. The duration of object exploration, which was defined as the mouse’s nose being directed towards the object while it was situated at a distance < 2 cm from the object, was scored off-line for each object and both objects combined. A percentage of time of exploration for the “new object” on day 2 that had replaced the “familiar object” from day 1 over the total duration of exploration was compared against a 50%-chance level of approaching either object and was taken as a parameter of object recognition memory.

#### 4.6.7. Fear Conditioning

The contextual fear conditioning test procedure was adapted from previously described protocols [210]. The apparatus consisted of a transparent plastic cubicle (25 cm × 25 cm × 50 cm) with a stainless-steel grid floor (33 rods 2 mm in diameter). A shocker was used to deliver an alternating electric current (AC, 50 Hz; 0.7 mA, 2 s) after a 2 min acclimatization of a mouse to the chamber. After delivery of the current, the mouse was immediately placed back in the home cage. Freezing behavior was scored by visual observation during a test of memory recall that was carried out 24 h later. The occurrence of freezing behavior in the chamber was assessed every 10 s for 180 s; each 10 s score was assigned to a freezing or non-freezing period, and the percentage of time spent in freezing was calculated.

### 4.7. Quantitative Real-Time qPCR

Total mRNA was extracted from each brain region using the RNeasy Lipid Tissue Mini Kit (Qiagen, Hilden, Germany). First-strand cDNA synthesis was performed using 1 μg of total RNA and the QuantiTect Reverse Transcription Kit (Qiagen, Hilden, Germany). qRT-PCR was conducted using SYBR Green Master Mix (Bio-Rad Laboratories, Philadelphia, PA, USA) on a ProFlex qPCR system (Thermo Fisher Scientific, Waltham, MA, USA). Each 10 μL reaction contained 5 μL SYBR Green master mix, 3 μL RNase-free water, 1 μL of specific forward and reverse primers (20 pmol/μL), and 1 μL cDNA. GAPDH was used as a reference gene, since it showed consistent stability across multiple paradigms and suggested by RefFinder analysis [207]. The qRT-PCR protocol consisted of an initial denaturation at 95 °C for 4 min, followed by 40 cycles of denaturation at 95 °C for 20 s and annealing at 54 °C for 90 s. Primer sequences are provided in Supplementary file, Table S5. All samples were analyzed in triplicate. Gene expression was normalized to GAPDH and calculated as relative fold changes using previously established methods [208,209,210,211,217,218,219].

### 4.8. A Study of the Effects DDW on the Protonophore FCCP-Induced Ca^2+^ Influx, a Model of Neurotoxicity and Neuronal Damage, in a Rat Neuronal Culture

**Figure 7:**
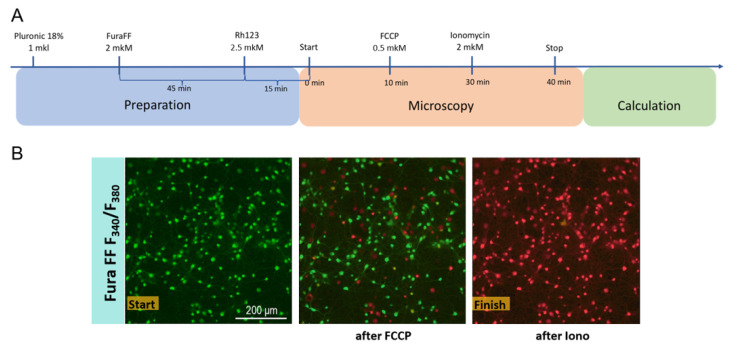
Study flow with FCCP-induced model of Ca^2+^-dependent neurotoxicity and representative fluorescent images of Ca^2+^ influx in a rat neuronal culture. (**A**) A study of the effects of decreased deuterium levels on the protonophore FCCP-induced Ca^2+^ influx, a model of neurotoxicity and neuronal damage, in a rat neuroglial culture hippocampus. (**B**) Representative fluorescent images of Ca^2+^ influx in a rat neuronal culture at the start of the experiment, and after FCCP and Ionomycin (Iono) treatment.

To address potential effects of DDW on mitochondrial functions, we employed Ca^2+^-dependent neurotoxicity model of oxidative stress in neuroglial cell culture of rat brain (Figure 7A). Wistar rat pups (P1–P2) were used for preparation of a primary mixed cortical neuronal culture. Cells were dissociated and suspended by pipetting with papain and then washed by gradient sedimentation in centrifuge. After collecting the cellular fraction, cells were stained with Trypan Blue for counting of live cells. Cells were seeded in Petri dishes that were pre-coated with polyethyleneimine and then incubated for 10–12 days. The experimental culture was incubated in a buffer with added DDW of 5 ppm for 30 min. Control cultures were left in regular buffer. By the end of culturing, in cell culture remained as control, buffer had unchanged deuterium levels, while cell culture exposed to DDW-containing buffer had a decrease in deuterium levels by 20 ppm (constituted 120 ppm) in a medium. For the neurotoxicity challenge, FCCP protonophore was used as described elsewhere [220]. Intracellular Ca^2+^ concentration and mitochondrial potential were measured using fluorescent microscopy. Four independent experiments were performed.

Fluorescent measurement of mitochondrial potential and intracellular Ca^2+^ concentration were performed in control and DDW-exposed cell culture samples. First, plates were treated with 1 μL 18% Pluronic in order to ensure delivery of the fluorescent dyes into the intracellular compartments. Thereafter, 45 min before the beginning of measurements, cells were treated with 2 μM acetoxymethyl ester of low-affinity Ca^2+^ indicator Fura-FF (Fura-FF/AM), and 15 min prior to the beginning of measurements, cells were loaded with 2.5 μM potential-sensitive dye Rhodamine 123 (Rh123). Ten minutes after the onset of measurement, 0.5 μM FCCP was added to induce mitochondrial Ca^2+^ release, a marker of calcium-dependent neurotoxicity, per-cell outcomes (AUC, time) were recorded. After 20 min of the measurement 2μM Ionomycin was added and the measurement was continued for another 10 min so the maximal Ca^2+^ cell capacities (calcium plateau) could be evaluated (Figure 7B).

### 4.9. Statistical Analysis

Data were analyzed using the statistical software package GraphPad PRISM 9.1.0 (GraphPad Software, San Diego, CA, USA). No criteria were set for including and excluding animals. The group sizes in the study were determined by the initial assessment of the statistical power calculated for the assays employed in the study. The Kolmogorov–Smirnov test was employed to assess the normality of the data distribution. For data exhibiting normal distribution, unpaired Fisher’s *t*-test and repeated measures two-way ANOVA were utilized as appropriate. The Geisser–Greenhouse correction was applied to the repeated measures ANOVA, and Šídák’s multiple comparison test was conducted for post hoc analysis. The Mann–Whitney test was employed for data not conforming to normal distribution. One-sample *t*-test was used for comparison with chance level. In the cell culture assay, in each of the four independent experiments (one CW and one DDW dish per experiment), the number of cells reaching the calcium plateau was determined, and the values were compared between CW- and DDW-based cell maintenance using the Mann–Whitney U-test. Per-cell outcomes were analyzed using robust linear mixed-effects models to account for the hierarchical structure of the data (cells nested within independent experiments) and to provide reliable inferences under potential deviations from Gaussian assumptions. In Wilkinson notation, outcome ~ condition + (1|experiment). The fixed-effect condition distinguished between CW and DDW treatments, and the random intercept experiment accounted for the correlation of cells measured within the same experiment. Each experiment corresponded to one of the four independent pairs of culture dish. Models were fitted using the rlmer function from the robustlmm R package (v3.3-3; https://cran.r-project.org/package=robustlmm, accessed on 22 September 2025). The significance level was established at 95% (*p* < 0.05). No data points were excluded from analysis. Data are presented as mean ± SEM. Group sizes and cell counts are indicated in figure legends unless stated in the Section 4.

## 5. Conclusions

In conclusion, in a mouse model of LLD, there were modest but significant changes in the gene expression of markers of plasticity, inflammation, and cellular stress. Reduced deuterium intake exerted limited but significant effects on LLD-related behaviors and brain gene expression in 18-month-old mice. Decreased deuterium levels were associated with increased resilience of neuronal primary cell culture to Ca^2+^-induced toxicity. qPCR assay supported changes of several genes highlighted in the Illumina experiments. These gene expression and mitochondrial effects could potentially underpin LLD-related symptoms in aged mice and effects of DDW on these animals, while numerous epigenetic, posttranslational, and other regulatory processes are likely to be involved in reported physiological changes as well. In sum, our findings suggest a physiological link between geographical factors that determine the isotopic content of drinking water and the risk of human morbidities associated with aging, such as LLD. In this context, the demonstrated significant effects of naturally occurring deuterium concentrations in drinking water raise important questions regarding the adequacy of current water standards.

## Figures and Tables

**Table 1:** Gene expression profiling of the hippocampi and prefrontal cortex of old vs. young mice. Genes with negative fold change are highlighted in red. The majority of altered genes were found in the prefrontal cortex.

	|Fold Change| ≥ 2.0	|Fold Change| ≥ 2.5
Hippocampus	Hba-a1, Ccl21a, Xlr4a, Erdr1, Ccl21c, Pik3r3, Cirbp	Hba-a1, Ccl21a, Xlr4a, Erdr1, Ccl21c
Prefrontal cortex	Hba-a1, Ccl21a, Stk32c, Ccl21c, Fus, Dynll2, Hbb-b1, Erdr1, Stmn4, Islr2, Lsm14b, Bax, Vapb, Pik3r3, Metrn, Chmp4b, Xlr4a, Prr7, Egr2, Dlg4, Atp1a2, Gpr17, Zxda, Rhbdl2, Prf1, Chka, Snhg11, Col6a1, Stxbp2, Negr1, Dpy30, Cd59a	Hba-a1, Ccl21a, Stk32c, Ccl21c, Fus, Dynll2, Hbb-b1, Erdr1, Zxda, Rhbdl2, Prf1, Chka, Snhg11

**Table 2:** Functional roles of hippocampal genes whose expression in aged mice was altered ≥ 2.0-fold vs. young mice. In the hippocampi of the group of older mice, one gene was significantly upregulated, whereas six genes were significantly downregulated, each exceeding a 2.0-fold threshold compared to the young animals. These genes are implicated in inflammation, cellular stress, synaptic plasticity, emotionality, and regeneration and are known to be altered in neurological disorders and aging. Overlapping changes in the prefrontal cortex (five genes) are shown in red.

ID of Gene	Gene Name	Old vs. Young Mice, Fold Change	Functional Role of Encoded Protein
*Hba-a1*	Hemoglobin subunit alpha-1	−4.97	Oxygen transport and oxidative metabolism [84]; beta-thalassemia [85].
*Ccl21a*	C-C motif chemokine 21a	−4.37	Immune cell trafficking, initial recruitment of T cells, balance of immune responses in lymphoid and nonlymphoid tissues [86].
*Ccl21c*	C-C motif chemokine 21c	−2.66	
*Erdr1*	Erythroid differentiation regulator 1	−2.89	Cell survival under stress, cellular growth, activation of immune system [87]; anti-inflammatory cytokine production/inhibition in macrophages [88].
*Xlr4a*	X-linked lymphocyte-regulated 4A	−3.40	Chromatin remodeling and dendritic spine morphology [89].
*Pik3r3*	Phosphoinositide-3-kinase regulatory subunit 3	−2.12	Cellular growth and proliferation in cancer [90], PPARα-mediated hepatic lipid metabolism [91].
*Cirbp*	Cold-inducible RNA binding protein	2.10	RNA binding, stress response [92,93].

**Table 3:** Functional roles of genes in the prefrontal cortex whose expression in old mice was changed ≥ 2.0-fold vs. young mice. In the prefrontal cortex of old mice, 22 genes were significantly downregulated and 10 genes were significantly upregulated, each exceeding a 2-fold threshold compared to the young group. These genes are implicated in cell growth, differentiation, survival, cancer, and stress responses. Overlapping changes in the hippocampus are indicated in red.

ID of Gene	Gene Name	Old vs. Young Mice, Fold Change	Functional Role of Encoded Protein
*Zxda*	Zinc finger X-linked duplicated A	4.36	Histocompatibility complex class I and class II regulation [94].
*Rhbdl2*	Rhomboid-like 2	4.25	Cleavage of thrombomodulin; wound healing [95], proliferation, migration, and invasion of pancreatic cancer [96].
*Prf1*	Perforin-1	3.96	Cytotoxic granule-mediated killing of infected or malignant cells [97], T-cell proliferation and cytokine production [98].
*Chka*	Choline kinase alpha	3.19	Tumor growth, cell proliferation mediated via EGFR/PI3K/AKT signaling [99].
*Snhg11*	Small nucleolar RNA host gene 11	2.35	Cancer cell proliferation, invasion and migration (glioma, lung cancer, and gastric cancer) [100,101].
*Col6a1*	Collagen type VI alpha 1 chain	2.19	Component of type VI collagen, autophagy, and apoptosis in megakaryocytes and neurons [102,103,104,105]; muscle regeneration [106].
*Stxbp2*	Syntaxin-binding protein 2	2.14	Synaptic transmission, neurodevelopment [107], NK-cell and T-cell functions [108].
*Negr1*	Neuronal growth regulator 1	2.12	Neuronal growth, synapse formation, and neurotransmission [109,110].
*Dpy30*	Protein dpy-30 homolog	2.07	Methylation of histone H3, early embryonic development, and cell differentiation [111].
*Cd59a*	CD59a glycoprotein	2.07	Complement system, T-cell activity, and inflammation [112,113].
*Hba-a1*	Hemoglobin subunit alpha-1	−11.55	See Table 2
*Hbb-b1*	Hemoglobin subunit beta-1	−3.16	Oxygen transport [84], beta-thalassemia [85].
*Ccl21a*	C-C motif chemokine 21a	−4.68	See Table 2
*Ccl21c*	C-C motif chemokine 21c	−3.25	
*Stk32c*	Serine/threonine kinase 32C	−3.87	Bladder and breast cancer, tumor cell proliferation, migration, and invasion [114,115].
*Fus*	RNA-binding protein fused in sarcoma	−3.19	DNA/RNA-binding, RNA transcription, splicing and transport, DNA repair, amyotrophic lateral sclerosis [116,117].
*Dynll2*	Dynein light chain 2	−3.17	Intracellular retrograde transport of vesicles and organelles [118,119].
*Erdr1*	Erythroid differentiation regulator 1	−2.80	See Table 2
*Stmn4*	Stathmin-4	−2.46	Adult hippocampal neurogenesis and spinogenesis [120,121]; demyelinating disorders, myelin repair [122].
*Islr2*	Immunoglobulin superfamily containing leucine-rich repeat 2	−2.33	Axon guidance, brain development [123]; peripheral nervous system and forebrain connectivity [124].
*Lsm14b*	LSM14 homolog B	−2.28	RNA-binding, oocyte maturation and early development; storage, stability, and translation of maternal mRNAs [125,126,127].
*Bax*	BCL2-associated X protein	−2.24	Programmed cell death, activation of the intrinsic apoptotic pathway, development and response to cellular stress [128].
*Vapb*	Vesicle-associated membrane protein-associated protein B	−2.22	Membrane trafficking, lipid transport, and membrane contacts; cellular lipid homeostasis [129,130].
*Pik3r3*	Phosphoinositide-3-kinase regulatory subunit 3	−2.21	See Table 2
*Metrn*	Meteorin	−2.17	Glial cell specialization and axonal development, neurogenesis [131].
*Chmp4b*	Charged multivesicular body protein 4B	−2.13	Component of the endosomal sorting complex transport-III, endosomal sorting, neuronal apoptosis [132], cell proliferation in hepatocellular carcinoma [133].
*Xlr4a*	X-linked lymphocyte-regulated 4A	−2.13	See Table 2
*Prr7*	Proline-rich 7	−2.11	T-cell receptor signaling and apoptosis [134,135], T-cell development [136]; synaptic depression [137].
*Egr2*	Early growth response 2	−2.10	DNA-binding transcription factor [138]; myelination in the peripheral nervous system and Schwann cell differentiation [139,140].
*Dlg4*	Disks large homolog 4	−2.09	Synaptogenesis and synaptic plasticity [141,142,143]; microglia development and inflammatory responses [144].
*Atp1a2*	ATPase Na+/K+ transporting subunit alpha 2	−2.05	Catalytic component of ATPase Na+/K+, brain development [145,146].

**Table 4:** Gene expression profiling of the hippocampi and prefrontal cortex of old mice exposed to DDW. Genes with negative fold change are highlighted in red. The majority of altered gene was in the hippocampus. All *n* = 20.

	|Fold Change| ≥ 1.25
Hippocampus	*Prdx4*, *Prkd3*, *Alkbh3*, *Ccno*, *Egr1*, *Homer1*, *Rbm28*, *Gadd45a*, *Rngtt*
Prefrontal cortex	*Crmp1*, *Dbp*, *Galnt9*, *Egr1*, *Sgsm1*, *Per2*

**Table 5:** Functional roles of genes whose expression in the hippocampus of the DDW group was altered ≥ 1.25-fold vs. CW mice. In the hippocampi of the DDW group, five genes were significantly upregulated, while four genes were significantly downregulated, each exceeding a 1.25-fold threshold compared to the CW group. These genes are implicated in cellular stress, plasticity, and regeneration and are known to be altered in neurological disorders and aging.

ID of Gene	Gene Name	DDW Group Fold- Change	Functional Role of Encoded Protein
*Egr1*	Early Growth Response 1	1.52	Memory consolidation and reconsolidation [147,148,149,150].
*Homer1*	Homer Scaffold Protein 1	1.43	Postsynaptic density and mGluR signaling [151,152], clinical depression [153].
*Rbm28*	RNA-binding protein 28	1.27	DNA repair, cellular stress response [154].
*Gadd45a*	Growth Arrest And DNA Damage Inducible Alpha	1.26	DNA repair, neuronal survival [155]; mitogenesis [156].
*Rngtt*	RNA Guanylyltransferase And 5′-Phosphatase	1.26	mRNA protection and transport [157].
*Prdx4*	Peroxiredoxin 4	−1.21	Oxidative damage [158,159], neurogenesis and neuronal differentiation [160].
*Prkd3*	Protein Kinase D3	−1.27	Cell signaling and growth, protein transport and transcription; cancer cell proliferation, growth, migration and invasion [161]
*Alkbh3*	AlkB homolog 3	−1.27	DNA and RNA repair, cell survival and cancer [162,163,164].
*Ccno*	Cyclin O	−1.32	Deuterosome formation and centriole amplification in multiciliated cells [165].

**Table 6:** Genes of the prefrontal cortex whose expression in DDW group was altered ≥ 1.25-fold vs. CW mice and their function. In the prefrontal cortex of the DDW group, four genes were significantly upregulated, while two genes were significantly downregulated by more than 1.25-fold compared to the CW group. These genes are implicated in the mechanisms of stress, plasticity, and circadian rhythms and are altered in neurological disorders and aging.

ID of Gene	Gene Name	DDW Group Fold- Change	Functional Role of Encoded Protein
*Galnt9*	Polypeptide N-Acetylgalactos-aminyltransferase 9	1.66	O-linked oligosaccharide biosynthesis, mitoprotective effects [166].
*Egr1*	Early Growth Response 1	1.61	See Table 1
*Sgsm1*	Small G Protein Signaling Modulator 1	1.35	Brain functions [167]; neuro-oncological disorders [168].
*Per2*	Period Circadian Regulator 2	−1.33	Circadian regulation [169]; major depression [170]; other psychotic disorders [171].
*Crmp1*	Collapsin Response Mediator Protein 1	−1.28	Neuronal development and axonal guidance [172]; neurodevelopmental disorders [173].
*Dbp*	Albumin D-site-Binding Protein	1.30	Circadian regulation [174,175].

**Table 7:** GO-BP pathways significantly changed in the hippocampi of the DDW group. Two GO-BP pathways were significantly altered in the prefrontal cortex in the DDW-exposed aged mice: “Adult walking behavior” and “Chaperone-mediated protein folding”.

GO-BP Term	Pathway Description	FDR-Corrected *p*-Value	NES
GO:0099645	Neurotransmitter receptor localization to postsynaptic specialization membrane	0.001	0.001
GO:0007268	Chemical synaptic transmission	0.023	0.023
GO:0007214	Gamma-aminobutyric acid signaling pathway	0.029	0.029
GO:0007616	Long-term memory	0.029	0.029
GO:0050773	Regulation of dendrite development	0.029	0.029
GO:0007399	Nervous system development	0.034	0.034
GO:0099175	Regulation of postsynapse organization	0.039	0.039
GO:0016477	Cell migration	0.039	0.039

**Table 8:** KEGG pathways significantly changed in the hippocampi of the DDW group. Three KEGG pathways were significantly altered in the prefrontal cortex in the DDW-exposed old animals: “AGE-RAGE signaling pathway in diabetic complications”, “FoxO signaling pathway”, and “Human T-cell leukemia virus 1 infection”.

GO-BP Term	Pathway Description	FDR-Corrected *p*-Value	NES
mmu04810	Regulation of actin cytoskeleton	0.004	1.76
mmu04921	Oxytocin signaling pathway	0.004	1.83
mmu04024	cAMP signaling pathway	0.005	1.70
mmu04022	cGMP-PKG signaling pathway	0.005	1.75
mmu04148	Efferocytosis	0.005	1.75
mmu04961	Endocrine and other factor-regulated calcium reabsorption	0.007	1.91
mmu04142	Lysosome	0.009	−1.73
mmu04010	MAPK signaling pathway	0.009	1.57
mmu04724	Glutamatergic synapse	0.009	1.78
mmu05202	Transcriptional misregulation in cancer	0.009	1.66
mmu05031	Amphetamine addiction	0.009	1.85
mmu04261	Adrenergic signaling in cardiomyocytes	0.011	1.66
mmu05030	Cocaine addiction	0.012	1.88
mmu04144	Endocytosis	0.015	1.57
mmu05032	Morphine addiction	0.015	1.76
mmu04510	Focal adhesion	0.023	1.57
mmu04068	FoxO signaling pathway	0.031	1.61
mmu04928	Parathyroid hormone synthesis, secretion, and action	0.031	1.64
mmu00970	Aminoacyl-tRNA biosynthesis	0.042	−1.81
mmu04020	Calcium signaling pathway	0.042	1.47
mmu04977	Vitamin digestion and absorption	0.042	−1.81
mmu04713	Circadian entrainment	0.042	1.62
mmu04666	Fc gamma R-mediated phagocytosis	0.048	1.61
mmu04962	Vasopressin-regulated water reabsorption	0.048	1.73
mmu05135	Yersinia infection	0.048	1.55

## Data Availability

The raw data supporting the conclusions of this article will be made available by the authors on request.

## Supplementary Materials

The following supporting information can be downloaded at https://www.mdpi.com/article/10.3390/ijms262110626/s1.

## References

[1] Katon W.J., Schoenbaum M., Fan M.-Y., Callahan C.M., Williams J., Hunkeler E., Harpole L., Zhou X.-H.A., Langston C., Unützer J. (2005). Cost-Effectiveness of Improving Primary Care Treatment of Late-Life Depression. Arch. Gen. Psychiatry.

[2] Smit F., Ederveen A., Cuijpers P., Deeg D., Beekman A. (2006). Opportunities for Cost-Effective Prevention of Late-Life Depression: An Epidemiological Approach. Arch. Gen. Psychiatry.

[3] Luppa M., Sikorski C., Motzek T., Konnopka A., König H.-H., Riedel-Heller S.G. (2012). Health Service Utilization and Costs of Depressive Symptoms in Late Life—A Systematic Review. Curr. Pharm. Des..

[4] Bock J.-O., Brettschneider C., Weyerer S., Werle J., Wagner M., Maier W., Scherer M., Kaduszkiewicz H., Wiese B., Moor L. (2016). Excess Health Care Costs of Late-Life Depression—Results of the AgeMooDe Study. J. Affect. Disord..

[5] Holvast F., Massoudi B., Oude Voshaar R.C., Verhaak P.F.M. (2017). Non-Pharmacological Treatment for Depressed Older Patients in Primary Care: A Systematic Review and Meta-Analysis. PLoS ONE.

[6] Naismith S.L., Norrie L.M., Mowszowski L., Hickie I.B. (2012). The Neurobiology of Depression in Later-Life: Clinical, Neuropsychological, Neuroimaging and Pathophysiological Features. Prog. Neurobiol..

[7] Cui X., Lyness J.M., Tang W., Tu X., Conwell Y. (2008). Outcomes and Predictors of Late-Life Depression Trajectories in Older Primary Care Patients. Am. J. Geriatr. Psychiatry.

[8] Butters M.A., Whyte E.M., Nebes R.D., Begley A.E., Dew M.A., Mulsant B.H., Zmuda M.D., Bhalla R., Meltzer C.C., Pollock B.G. (2004). The Nature and Determinants of Neuropsychological Functioning in Late-Life Depression. Arch. Gen. Psychiatry.

[9] Alexopoulos G.S. (2005). Depression in the Elderly. Lancet.

[10] Hegeman J.M., Kok R.M., van der Mast R.C., Giltay E.J. (2012). Phenomenology of Depression in Older Compared with Younger Adults: Meta-Analysis. Br. J. Psychiatry.

[11] Teixeira A.L., Dumas J.A. (2022). Further Evidence of Peripheral Inflammatory Markers Involvement in Late-Onset Depression and Cognitive Decline. Am. J. Geriatr. Psychiatry.

[12] Diniz B.S., Teixeira A.L., Talib L.L., Mendonça V.A., Gattaz W.F., Forlenza O.V. (2010). Serum Brain-Derived Neurotrophic Factor Level Is Reduced in Antidepressant-Free Patients with Late-Life Depression. World J. Biol. Psychiatry.

[13] Taylor W.D., Aizenstein H.J., Alexopoulos G.S. (2013). The Vascular Depression Hypothesis: Mechanisms Linking Vascular Disease with Depression. Mol. Psychiatry.

[14] Bruno D., Reichert Plaska C., Clark D.P.A., Zetterberg H., Blennow K., Verbeek M.M., Pomara N. (2021). CSF α-Synuclein Correlates with CSF Neurogranin in Late-Life Depression. Int. J. Neurosci..

[15] Blaveri E., Kelly F., Mallei A., Harris K., Taylor A., Reid J., Razzoli M., Carboni L., Piubelli C., Musazzi L. (2010). Expression Profiling of a Genetic Animal Model of Depression Reveals Novel Molecular Pathways Underlying Depressive-like Behaviours. PLoS ONE.

[16] Marchetti L., Lauria M., Caberlotto L., Musazzi L., Popoli M., Mathé A.A., Domenici E., Carboni L. (2020). Gene Expression Signature of Antidepressant Treatment Response/Non-Response in Flinders Sensitive Line Rats Subjected to Maternal Separation. Eur. Neuropsychopharmacol..

[17] Hamilton M. (1967). Development of a rating scale for primary depressive illness. Br. J. Soc. Clin. Psychol..

[18] Mangold C.A., Wronowski B., Du M., Masser D.R., Hadad N., Bixler G.V., Brucklacher R.M., Ford M.M., Sonntag W.E., Freeman W.M. (2017). Sexually Divergent Induction of Microglial-Associated Neuroinflammation with Hippocampal Aging. J. Neuroinflamm..

[19] Li M., Su S., Cai W., Cao J., Miao X., Zang W., Gao S., Xu Y., Yang J., Tao Y.-X. (2020). Differentially Expressed Genes in the Brain of Aging Mice with Cognitive Alteration and Depression- and Anxiety-Like Behaviors. Front. Cell Dev. Biol..

[20] Kang H.J., Voleti B., Hajszan T., Rajkowska G., Stockmeier C.A., Licznerski P., Lepack A., Majik M.S., Jeong L.S., Banasr M. (2012). Decreased Expression of Synapse-Related Genes and Loss of Synapses in Major Depressive Disorder. Nat. Med..

[21] Zhao J., Verwer R.W.H., Gao S.-F., Qi X.-R., Lucassen P.J., Kessels H.W., Swaab D.F. (2018). Prefrontal Alterations in GABAergic and Glutamatergic Gene Expression in Relation to Depression and Suicide. J. Psychiatr. Res..

[22] Zeng L., Fujita M., Gao Z., White C.C., Green G.S., Habib N., Menon V., Bennett D.A., Boyle P., Klein H.-U. (2024). A Single-Nucleus Transcriptome-Wide Association Study Implicates Novel Genes in Depression Pathogenesis. Biol. Psychiatry.

[23] Nagy C., Schwabe D., Jones W., Brown A., Shupe M., Mancone A., Camillocci J. (2016). “Time to Talk About It: Physician Depression and Suicide” Video/Discussion Session for Interns, Residents, and Fellows. MedEdPORTAL.

[24] Primiani C.T., Ryan V.H., Rao J.S., Cam M.C., Ahn K., Modi H.R., Rapoport S.I. (2014). Coordinated Gene Expression of Neuroinflammatory and Cell Signaling Markers in Dorsolateral Prefrontal Cortex during Human Brain Development and Aging. PLoS ONE.

[25] De Sousa R.A.L., Rocha-Dias I., de Oliveira L.R.S., Improta-Caria A.C., Monteiro R.S., Cassilhas R.C. (2021). Molecular mechanisms of physical exercise on depression in the elderly: A systematic review. Mol. Biol. Rep..

[26] Cardona D., Segura A., Segura Á., Garzón M.O. (2015). Contextual effects associated with depression risk variability in the elderly, Antioquia, Colombia, 2012. Biomedica.

[27] Saravanakumar P., Muhammad T., Paul R., Srivastava S. (2024). Explaining the Urban-Rural Difference in Late-Life Depression in India: Evidence from a Multivariate Decomposition Analysis Based on Longitudinal Aging Study in India, Wave 2017–2018. Clin. Gerontol..

[28] Behlen M., Henriques A., Severo M., Santos C.J., Ribeiro A.I. (2023). Exposure to Green and Blue Spaces and Depression in Older Adults: Findings from the EPIPorto Cohort. Eur. J. Public Health.

[29] D’Souza J., Bergmans R., Fossa A., Szpiro A.A., Mendes de Leon C., Kaufman J., Hirth R., Faul J., Adar S. Physical Environmental Hazards and Major Depression in Older U.S. Adults: The Health and Retirement Study. Proceedings of the ISEE 2022: 34th Annual Conference of the International Society of Environmental Epidemiology.

[30] Bauer M., Glenn T., Alda M., Andreassen O.A., Ardau R., Bellivier F., Berk M., Bjella T.D., Bossini L., Del Zompo M. (2012). Impact of Sunlight on the Age of Onset of Bipolar Disorder. Bipolar Disord..

[31] Li X., Su H., Xia Y., Zhao Y. (2021). The Association between Water Source and Depressive Symptoms in China: A Cross-Sectional and Longitudinal Study. J. Affect. Disord..

[32] Ljubicić D., Stipcević T., Pivac N., Jakovljević M., Mück-Seler D. (2007). The Influence of Daylight Exposure on Platelet 5-HT Levels in Patients with Major Depression and Schizophrenia. J. Photochem. Photobiol. B.

[33] Komulainen K., Hakulinen C., Lipsanen J., Partonen T., Pulkki-Råback L., Kähönen M., Virtanen M., Ruuhela R., Raitakari O., Elovainio M. (2022). Associations of Long-Term Solar Insolation with Specific Depressive Symptoms: Evidence from a Prospective Cohort Study. J. Psychiatr. Res..

[34] Maes M., De Meyer F., Thompson P., Peeters D., Cosyns P. (1994). Synchronized Annual Rhythms in Violent Suicide Rate, Ambient Temperature and the Light-Dark Span. Acta Psychiatr. Scand..

[35] Brazienė A., Venclovienė J., Vaičiulis V., Lukšienė D., Tamošiūnas A., Milvidaitė I., Radišauskas R., Bobak M. (2022). Relationship between Depressive Symptoms and Weather Conditions. Int. J. Environ. Res. Public Health.

[36] Flaten T.P. (2001). Aluminium as a Risk Factor in Alzheimer’s Disease, with Emphasis on Drinking Water. Brain Res. Bull..

[37] Brown J.S. (1994). Role of Selenium and Other Trace Elements in the Geography of Schizophrenia. Schizophr. Bull..

[38] Baj J., Bargieł J., Cabaj J., Skierkowski B., Hunek G., Portincasa P., Flieger J., Smoleń A. (2023). Trace Elements Levels in Major Depressive Disorder-Evaluation of Potential Threats and Possible Therapeutic Approaches. Int. Mol. Sci..

[39] Heidari H., Lawrence D.A. (2023). Climate Stressors and Physiological Dysregulations: Mechanistic Connections to Pathologies. Int. J. Environ. Res. Public Health.

[40] Sundas A., Contreras I., Mujahid O., Beneyto A., Vehi J. (2024). The Effects of Environmental Factors on General Human Health: A Scoping Review. Healthcare.

[41] Alum E.U. (2024). Climate Change and Its Impact on the Bioactive Compound Profile of Medicinal Plants: Implications for Global Health. Plant Signal. Behav..

[42] Dutton A., Wilkinson B.H., Welker J.M., Bowen G.J., Lohmann K.C. (2005). Spatial Distribution and Seasonal Variation in^18^O/^16^O of Modern Precipitation and River Water across the Conterminous USA. Hydrol. Process..

[43] Kendall C., Coplen T.B. (2001). Distribution of Oxygen--18 and Deuterium in River Waters across the United States. Hydrol. Process..

[44] Friedman I., Redfield A.C., Schoen B., Harris J. (1964). The Variation of the Deuterium Content of Natural Waters in the Hydrologic Cycle. Rev. Geophys..

[45] International Atomic Energy Agency (2017). Reference Sheet for VSMOW2 and SLAP2.

[46] Strekalova T., Evans M., Chernopiatko A., Couch Y., Costa-Nunes J., Cespuglio R., Chesson L., Vignisse J., Steinbusch H.W., Anthony D.C. (2015). Deuterium Content of Water Increases Depression Susceptibility: The Potential Role of a Serotonin-Related Mechanism. Behav. Brain Res..

[47] Bowen G.J., Ehleringer J.R., Chesson L.A., Stange E., Cerling T.E. (2007). Stable Isotope Ratios of Tap Water in the Contiguous United States. Water Resour. Res..

[48] Goncharuk V.V., Lapshin V.B., Burdeinaya T.N., Pleteneva T.V., Chernopyatko A.S., Atamanenko I.D., Ul’Yantsev A.S., Uspenskaya E.V., Samsoni-Todorov A.O., Taranov V.V. (2011). Physicochemical properties and biological activity of the water depleted of heavy isotopes. J. Water Chem. Technol..

[49] Gat J.R., Magaritz M. (1980). Climatic Variations in the Eastern Mediterranean Sea Area. Naturwissenschaften.

[50] Qu J., Xu Y., Zhao S., Xiong L., Jing J., Lui S., Huang J., Shi H. (2024). The Biological Impact of Deuterium and Therapeutic Potential of Deuterium-Depleted Water. Front. Pharmacol..

[51] Kirkina A.A., Lobyshev V.I., Lopina O.D., Doronin Y.-K., Burdeinaya T.N., Chernopyatko A.S. (2014). Isotopic effects of low concentration of deuterium in water on biological systems. Biofizika.

[52] Vasilescu V., Katona E. (1986). Deuteration as a Tool in Investigating the Role of Water in the Structure and Function of Excitable Membranes. Methods in Enzymology.

[53] Kravtsov A., Kozin S., Basov A., Butina E., Baryshev M., Malyshko V., Moiseev A., Elkina A., Dzhimak S. (2021). Reduction of Deuterium Level Supports Resistance of Neurons to Glucose Deprivation and Hypoxia: Study in Cultures of Neurons and on Animals. Molecules.

[54] Pomytkin I.A., Kolesova O.E. (2006). Relationship between Natural Concentration of Heavy Water Isotopologs and Rate of H_2_O_2_ Generation by Mitochondria. Bull. Exp. Biol. Med..

[55] Zhang X., Gaetani M., Chernobrovkin A., Zubarev R.A. (2019). Anticancer Effect of Deuterium Depleted Water-Redox Disbalance Leads to Oxidative Stress. Mol. Cell. Proteomics.

[56] Olgun A. (2007). Biological Effects of Deuteronation: ATP Synthase as an Example. Theor. Biol. Med. Model..

[57] Yaglova N.V., Timokhina E.P., Obernikhin S.S., Yaglov V.V. (2023). Emerging Role of Deuterium/Protium Disbalance in Cell Cycle and Apoptosis. Int. J. Mol. Sci..

[58] Wu Y., Qin D., Yang H., Wang W., Xiao J., Zhou L., Fu H. (2020). Neuroprotective Effects of Deuterium-Depleted Water (DDW) Against H_2_O_2_-Induced Oxidative Stress in Differentiated PC12 Cells Through the PI3K/Akt Signaling Pathway. Neurochem. Res..

[59] Bayrak B.B., Kulak G.Y., Yanardag R., Yarat A. (2022). Short Term Deuterium Depletion in Drinking Water Reduced Tumor Induced Oxidative Stress in Mice Liver. Pathol.-Res. Pract..

[60] Korchinsky N., Davis A.M., Boros L.G. (2024). Nutritional Deuterium Depletion and Health: A Scoping Review. Metabolomics.

[61] Lu Y., Chen H. (2024). Deuterium-Depleted Water in Cancer Therapy: A Systematic Review of Clinical and Experimental Trials. Nutrients.

[62] Molnár M., Horváth K., Dankó T., Somlyai I., Kovács B.Z., Somlyai G. (2021). Deuterium-Depleted Water Stimulates GLUT4 Translocation in the Presence of Insulin, Which Leads to Decreased Blood Glucose Concentration. Mol. Cell. Biochem..

[63] Somlyai G., Somlyai I., Fórizs I., Czuppon G., Papp A., Molnár M. (2020). Effect of Systemic Subnormal Deuterium Level on Metabolic Syndrome Related and Other Blood Parameters in Humans: A Preliminary Study. Molecules.

[64] Boros L.G., Somlyai I., Kovács B.Z., Puskás L.G., Nagy L.I., Dux L., Farkas G., Somlyai G. (2021). Deuterium Depletion Inhibits Cell Proliferation, RNA and Nuclear Membrane Turnover to Enhance Survival in Pancreatic Cancer. Cancer Control.

[65] Cocchi M., Tonello L., Gabrielli F., Pregnolato M. (2011). Depression, Osteoporosis, Serotonin and Cell Membrane Viscosity Between Biology and Philosophical Anthropology. Ann. Gen. Psychiatry.

[66] Cespuglio R., Rousset C., Debilly G., Rochat C., Millan M.J. (2005). Acute Administration of the Novel Serotonin and Noradrenaline Reuptake Inhibitor, S33005, Markedly Modifies Sleep-Wake Cycle Architecture in the Rat. Psychopharmacology.

[67] Newberg A.B., Amsterdam J.D., Wintering N., Shults J. (2012). Low Brain Serotonin Transporter Binding in Major Depressive Disorder. Psychiatry Res. Neuroimaging.

[68] Zia A., Pourbagher-Shahri A.M., Farkhondeh T., Samarghandian S. (2021). Molecular and Cellular Pathways Contributing to Brain Aging. Behav. Brain Funct..

[69] Bartman S., Coppotelli G., Ross J.M. (2024). Mitochondrial Dysfunction: A Key Player in Brain Aging and Diseases. Curr. Issues Mol. Biol..

[70] Mastrobattista E., Lenze E.J., Reynolds C.F., Mulsant B.H., Wetherell J., Wu G.F., Blumberger D.M., Karp J.F., Butters M.A., Mendes-Silva A.P. (2023). Late-Life Depression Is Associated with Increased Levels of GDF-15, a Pro-Aging Mitokine. Am. J. Geriatr. Psychiatry.

[71] Bustamante-Barrientos F.A., Luque-Campos N., Araya M.J., Lara-Barba E., Solminihac J., Pradenas C., Molina L., Herrera-Luna Y., Utreras-Mendoza Y., Elizondo-Vega R. (2023). Mitochondrial Dysfunction in Neurodegenerative Disorders: Potential Therapeutic Application of Mitochondrial Transfer to Central Nervous System-Residing Cells. J. Transl. Med..

[72] Li J., Cui J., Tian Y. (2022). Neuron-Periphery Mitochondrial Stress Communication in Aging and Diseases. Life Med..

[73] Glaesmer H., Riedel-Heller S., Braehler E., Spangenberg L., Luppa M. (2011). Age- and Gender-Specific Prevalence and Risk Factors for Depressive Symptoms in the Elderly: A Population-Based Study. Int. Psychogeriatr..

[74] Zanni G.R., Wick J.Y. (2010). Understanding Suicide in the Elderly. The Consultant Pharmacist.

[75] Chang C.-C., Yu S.-C., McQuoid D.R., Messer D.F., Taylor W.D., Singh K., Boyd B.D., Krishnan K.R.R., MacFall J.R., Steffens D.C. (2011). Reduction of Dorsolateral Prefrontal Cortex Gray Matter in Late-Life Depression. Psychiatry Res..

[76] Sheline Y.I. (2011). Depression and the Hippocampus: Cause or Effect?. Biol. Psychiatry.

[77] Zhao Z., Taylor W.D., Styner M., Steffens D.C., Krishnan K.R.R., MacFall J.R. (2008). Hippocampus Shape Analysis and Late-Life Depression. PLoS ONE.

[78] Malatynska E., Steinbusch H.W.M., Redkozubova O., Bolkunov A., Kubatiev A., Yeritsyan N.B., Vignisse J., Bachurin S., Strekalova T. (2012). Anhedonic-like Traits and Lack of Affective Deficits in 18-Month-Old C57BL/6 Mice: Implications for Modeling Elderly Depression. Exp. Gerontol..

[79] Lawton M.P., Parmelee P.A., Katz I.R., Nesselroade J. (1996). Affective States in Normal and Depressed Older People. J. Gerontol. B Psychol. Sci. Soc. Sci..

[80] Ngan J.S.T., Chan W.C., Wong S.T., Wong C.S.M., Cheng C.P.W. (2023). Reward System in Late-Life Depression: A Cross-Sectional Case-Control Study. East. Asian Arch. Psychiatry.

[81] Strekalova T., Bahzenova N., Trofimov A., Schmitt-Böhrer A.G., Markova N., Grigoriev V., Zamoyski V., Serkova T., Redkozubova O., Vinogradova D. (2018). Pro-Neurogenic, Memory-Enhancing and Anti-Stress Effects of DF302, a Novel Fluorine Gamma-Carboline Derivative with Multi-Target Mechanism of Action. Mol. Neurobiol..

[82] Zhang X., Wang J., Zubarev R.A. (2020). Slight Deuterium Enrichment in Water Acts as an Antioxidant: Is Deuterium a Cell Growth Regulator?. Mol. Cell. Proteomics.

[83] Shikama K., Matsuoka A. (2003). Human Haemoglobin: A New Paradigm for Oxygen Binding Involving Two Types of Alphabeta Contacts. Eur. J. Biochem..

[84] Weiss M.J., Zhou S., Feng L., Gell D.A., Mackay J.P., Shi Y., Gow A.J. (2005). Role of Alpha-Hemoglobin-Stabilizing Protein in Normal Erythropoiesis and Beta-Thalassemia. Ann. N. Y. Acad. Sci..

[85] Lo J.C., Chin R.K., Lee Y., Kang H.-S., Wang Y., Weinstock J.V., Banks T., Ware C.F., Franzoso G., Fu Y.-X. (2003). Differential Regulation of CCL21 in Lymphoid/Nonlymphoid Tissues for Effectively Attracting T Cells to Peripheral Tissues. J. Clin. Investig..

[86] Bhadhprasit W., Kinoshita C., Matsumura N., Aoyama K. (2024). Erythroid Differentiation Regulator 1 as a Regulator of Neuronal GSH Synthesis. Antioxidants.

[87] Wang Y. (2024). Erdr1 Drives Macrophage Programming via Dynamic Interplay with YAP1 and Mid1. Immunohorizons.

[88] Di Segni M., D’ADdario S.L., Babicola L., Ielpo D., Iacono L.L., Andolina D., Accoto A., Luchetti A., Mancini C., Parisi C. (2020). Xlr4 as a New Candidate Gene Underlying Vulnerability to Cocaine Effects. Neuropharmacology.

[89] Chen Q., Sun X., Luo X., Wang J., Hu J., Feng Y. (2020). PIK3R3 Inhibits Cell Senescence through P53/P21 Signaling. Cell Death Dis..

[90] Yang X., Fu Y., Hu F., Luo X., Hu J., Wang J. (2018). PIK3R3 Regulates PPARα Expression to Stimulate Fatty Acid β-Oxidation and Decrease Hepatosteatosis. Exp. Mol. Med..

[91] Lujan D.A., Ochoa J.L., Hartley R.S. (2018). Cold-Inducible RNA Binding Protein in Cancer and Inflammation. Wiley Interdiscip. Rev. RNA.

[92] Zhong P., Peng J., Bian Z., Huang H. (2021). The Role of Cold Inducible RNA-Binding Protein in Cardiac Physiology and Diseases. Front. Pharmacol..

[93] Al-Kandari W., Koneni R., Navalgund V., Aleksandrova A., Jambunathan S., Fontes J.D. (2007). The Zinc Finger Proteins ZXDA and ZXDC Form a Complex That Binds CIITA and Regulates MHC II Gene Transcription. J. Mol. Biol..

[94] Cheng T.L., Wu Y.T., Lin H.Y., Hsu F.C., Liu S.K., Chang B.I., Chen W.S., Lai C.H., Shi G.Y., Wu H.L. (2011). Functions of Rhomboid Family Protease RHBDL2 and Thrombomodulin in Wound Healing. J. Investig. Dermatol..

[95] Chen S., Cai K., Zheng D., Liu Y., Li L., He Z., Sun C., Yu C. (2022). RHBDL2 Promotes the Proliferation, Migration, and Invasion of Pancreatic Cancer by Stabilizing the N1ICD via the OTUD7B and Activating the Notch Signaling Pathway. Cell Death Dis..

[96] Pipkin M.E., Rao A., Lichtenheld M.G. (2010). The Transcriptional Control of the Perforin Locus. Immunol. Rev..

[97] Revelo X.S., Tsai S., Lei H., Luck H., Ghazarian M., Tsui H., Shi S.Y., Schroer S., Luk C.T., Lin G.H.Y. (2015). Perforin Is a Novel Immune Regulator of Obesity-Related Insulin Resistance. Diabetes.

[98] Hu L., Wang R.-Y., Cai J., Feng D., Yang G.-Z., Xu Q.-G., Zhai Y.-X., Zhang Y., Zhou W.-P., Cai Q.-P. (2016). Overexpression of CHKA Contributes to Tumor Progression and Metastasis and Predicts Poor Prognosis in Colorectal Carcinoma. Oncotarget.

[99] Wu Q., Ma J., Wei J., Meng W., Wang Y., Shi M., Zhang Y., Liu Y., Wang H., Wang Y. (2020). LncRNA SNHG11 Promotes Gastric Cancer Progression by Activating the Wnt/β-Catenin Pathway and Oncogenic Autophagy. Front. Oncol..

[100] Abbonante V., Malara A., Chrisam M., Metti S., Soprano P., Semplicini C., Bello L., Bozzi V., Battiston M., Pecci A. (2022). Lack of COL6/collagen VI causes megakaryocyte dysfunction by impairing autophagy and inducing apoptosis. Autophagy.

[101] Urciuolo A., Quarta M., Morbidoni V., Gattazzo F., Molon S., Grumati P., Montemurro F., Tedesco F.S., Blaauw B., Cossu G. (2013). Collagen VI regulates satellite cell self-renewal and muscle regeneration. Nat. Commun..

[102] Geng Y.B., Xu C., Wang Y., Zhang L.W. (2020). Activating transcription factor 3-activated long noncoding RNA forkhead box P4-antisense RNA 1 aggravates colorectal cancer progression by regulating microRNA-423-5p/nucleus accumbens associated 1 axis. Eur. Rev. Med. Pharmacol. Sci..

[103] Wei Z.Y., Wang L.P., Gao D., Zhu L., Wu J.F., Shi J., Li Y.N., Tang X.D., Feng Y.M., Pan X.B. (2024). Bulk and single-cell RNA-seq analyses reveal canonical RNA editing associated with microglia homeostasis and its role in sepsis-associated encephalopathy. Neuroscience.

[104] Kapustin A.N., Tsakali S.S., Whitehead M., Chennell G., Wu M.Y., Molenaar C., Kutikhin A., Chen Y., Ahmad S., Bogdanov L. (2025). Matrix-associated extracellular vesicles modulate human smooth muscle cell adhesion and directionality by presenting collagen VI. Elife.

[105] Manon-Jensen T., Kjeld N.G., Karsdal M.A. (2016). Collagen-mediated hemostasis. J. Thromb. Haemost..

[106] Maffucci P., Filion C.A., Boisson B., Itan Y., Shang L., Casanova J.L., Puel A., Revy P., Cunningham-Rundles C. (2016). Genetic Diagnosis Using Whole Exome Sequencing in Common Variable Immunodeficiency. Front. Immunol..

[107] Hao Y., Zhang X., Zhang M., Tang Y., Wu J., Wang T., Wang X., Zhang Y., Wang Z., Wang Y. (2018). Bi-Allelic Mutations in STXBP2 Reveal a Complementary Role for STXBP1 in Cytotoxic Lymphocyte Killing. Front. Immunol..

[108] Cwetsch A.W., Pinto B., Savardi A., Cancedda L. (2018). In vivo methods for acute modulation of gene expression in the central nervous system. Prog. Neurobiol..

[109] Sungur A.O., Schwarting R.K.W. (2012). The role of the lateral septum in anxiety and locomotion. Behav. Brain Res..

[110] Jiang H., Shukla A., Wang X., Chen W.Y., Bernstein B.E., Roeder R.G. (2011). Role for Dpy-30 in ES cell-fate specification by regulation of H3K4 methylation within bivalent domains. Cell.

[111] Jiang H., Chang F.C., Ross A.E., Lee J., Nakayama K., Nakayama K., Desiderio S. (2005). Ubiquitylation of RAG-2 by Skp2-SCF links destruction of the V(D)J recombinase to the cell cycle. Mol. Cell.

[112] Bartoletti A., Cancedda L., Reid S.W., Tessarollo L., Porciatti V., Pizzorusso T., Maffei L. (2002). Heterozygous knock-out mice for brain-derived neurotrophic factor show a pathway-specific impairment of long-term potentiation but normal critical period for monocular deprivation. J. Neurosci..

[113] Matsuo T., Ohtsuki T., Ohtsuki T., Koga M., Kato N., Kato T. (2007). Functional polymorphisms in the upstream region of the GRIK4 gene are associated with increased risk of schizophrenia. Am. J. Hum. Genet..

[114] Zhou Y., Zhou B., Pache L., Chang M., Khodabakhshi A.H., Tanaseichuk O., Benner C., Chanda S.K. (2019). Metascape provides a biologist-oriented resource for the analysis of systems-level datasets. Nat. Commun..

[115] Murray D.T., Kato M., Lin Y., Thurber K.R., Hung I., McKnight S.L., Tycko R. (2017). Structure of FUS protein fibrils and its relevance to self-assembly and phase separation of low-complexity domains. Cell.

[116] Mackenzie I.R.A., Neumann M. (2017). Fused in Sarcoma Neuropathology in Neurodegenerative Disease. Cold Spring Harb. Perspect. Med..

[117] Toropova K., Zalyte R., Woolfson D.N., Roberts A.J. (2019). Structure of the dynein-2 complex and its assembly with intraflagellar transport trains. Nat. Struct. Mol. Biol..

[118] Hou Y., Qin H., Follit J.A., Pazour G.J., Rosenbaum J.L., Witman G.B. (2007). Functional analysis of an individual IFT protein: IFT46 is required for transport of outer dynein arms into flagella. J. Cell Biol..

[119] Martel G., Uchida S., Hevi C., Chevere-Torres I., Fuentes I., Park Y.J., Hafeez H., Yamagata H., Watanabe Y., Shumyatsky G.P. (2016). Genetic demonstration of a role for stathmin in adult hippocampal neurogenesis, spinogenesis, and NMDA receptor-dependent memory. J. Neurosci..

[120] Liedtke W., Leman E.E., Fyffe R.E.W., Raine C.S., Schubart U.K. (2002). Stathmin-deficient mice develop an age-dependent axonopathy of the central and peripheral nervous systems. Am. J. Pathol..

[121] Liu A., Muggironi M., Marin-Husstege M., Casaccia-Bonnefil P. (2005). Oligodendrocyte process outgrowth in G1 phase is regulated by Cdc42 activation and cyclin-dependent kinase inhibition. J. Neurosci..

[122] Panza P., Sitko A.A., Maischein H.M., Koch I., Flötenmeyer M., Wright G.J., Mandai K., Mason C.A., Söllner C. (2015). The LRR receptor Islr2 is required for retinal axon routing at the vertebrate optic chiasm. Development.

[123] Abudureyimu S., Asai N., Enomoto A., Weng L., Kobayashi H., Wang X., Chen C., Mii S., Takahashi M. (2018). Essential role of Linx/Islr2 in the development of the forebrain commissural system. Cell Rep..

[124] Li H., Zhao H., Yang C., Su R., Long M., Liu J., Shi L., Xue Y., Su Y.Q. (2023). LSM14B is an oocyte-specific RNA-binding protein indispensable for maternal mRNA metabolism and oocyte development in mice. Adv. Sci..

[125] Chen Q., Chen Y., Zheng Q. (2025). The RNA-binding protein LSM family regulating reproductive development via different RNA metabolism. Biochim. Biophys. Acta Mol. Basis Dis..

[126] Wang J., Choi J.M., Holehouse A.S., Lee H.O., Zhang X., Jahnel M., Maharana S., Lemaitre R., Pozniakovsky A., Drechsel D. (2018). A Molecular Grammar Governing the Driving Forces for Phase Separation of Prion-like RNA Binding Proteins. Cell.

[127] Zhang X., Yu Z., Yin L., Li Q., He S., Li H., Li J., Sheng L., Wu H., Chen H. (2025). BAX-mediated ammonia-driven cell death: A novel prognostic and therapeutic target in clear cell renal cell carcinoma. Hum. Genom..

[128] Jiao J., Liu W., Gao G., Yang H. (2025). Serine-129 phosphorylated α-synuclein drives mitochondrial dysfunction and calcium dysregulation in Parkinson’s disease model. Front. Aging Neurosci..

[129] Li M., Zhang Y., Yu G., Gu L., Zhu H., Feng S., Xiong X., Jian Z. (2024). Mitochondria-associated endoplasmic reticulum membranes tethering protein VAPB-PTPIP51 protects against ischemic stroke through inhibiting the activation of autophagy. CNS Neurosci. Ther..

[130] Schwieger J., Wei C., Munro G., Petersen K.A., Lenarz T., Scheper V. (2025). Concentration Dependent Effects of Human Cometin on Spiral Ganglion Neuron Survival and Neurite Outgrowth. Audiol. Neurootol.

[131] Zhao P., Li C., Chen B., Sun G., Chao H., Tu Y., Bao Z., Fan L., Du X., Ji J. (2020). Up-regulation of CHMP4B alleviates microglial necroptosis induced by traumatic brain injury. J. Cell Mol. Med..

[132] Zhou Y., Bennett T.M., Shiels A. (2019). A charged multivesicular body protein (CHMP4B) is required for lens growth and differentiation. Differentiation.

[133] Imamura M., Matsumoto H., Mannen H., Takeda S., Aoki Y. (2024). The R436Q missense mutation in WWP1 disrupts autoinhibition of its E3 ubiquitin ligase activity, leading to self-degradation and loss of function. In Vitro Cellular & Developmental Biology—Animal.

[134] Lee S.H., Shin S.M., Zhong P., Kim H.T., Kim D.I., Kim J.M., Heo W.D., Kim D.W., Yeo C.Y., Kim C.H. (2018). Reciprocal control of excitatory synapse numbers by Wnt and Wnt inhibitor PRR7 secreted on exosomes. Nat. Commun..

[135] Kravchick D.O., Karpova A., Hrdinka M., Lopez-Rojas J., Iacobas S., Carbonell A.U., Iacobas D.A., Kreutz M.R., Jordan B.A. (2016). Synaptonuclear messenger PRR7 inhibits c-Jun ubiquitination and regulates NMDA-mediated excitotoxicity. EMBO J..

[136] Inouye M.O., Colameo D., Ammann I., Winterer J., Schratt G. (2022). miR-329- and miR-495-mediated Prr7 down-regulation is required for homeostatic synaptic depression in rat hippocampal neurons. Life Sci. Alliance..

[137] Han S.H., Cho J.G., Park S.J., Shin Y.K., Hong Y.B., Han J.Y., Park H.T., Park J.I. (2025). Transcription Factors and Coregulators in Schwann Cell Differentiation, Myelination, and Remyelination: Implications for Peripheral Neuropathy. J. Neurosci. Res..

[138] Jouaud M., Gonnaud P.M., Richard L., Latour P., Ollagnon-Roman E., Sturtz F., Mathis S., Magy L., Vallat J.M. (2020). A de novo EGR2 variant, c.1232A>G (p.Asp411Gly), causes a severe early-onset Charcot-Marie-Tooth disease type 1D. Neuromuscul. Disord..

[139] Topilko P., Schneider-Maunoury S., Levi G., Baron-Van Evercooren A., Chennoufi A.B., Seitanidou T., Babinet C., Charnay P. (1994). Krox-20 controls myelination in the peripheral nervous system. Nature.

[140] Migaud M., Charlesworth P., Dempster M., Webster L.C., Watabe A.M., Makhinson M., He Y., Ramsay M.F., Morris R.G., Morrison J.H. (1998). Enhanced long-term potentiation and impaired learning in mice with mutant postsynaptic density-95 protein. Nature.

[141] He J., Bellini M., Xu J., Castleberry A.M., Hall R.A. (2004). PDZ domain-containing protein PIST regulates the intracellular trafficking and function of the metabotropic glutamate receptor 5. J. Biol. Chem..

[142] Freudenberg F. (2019). Quantitative analysis of Gria1, Gria2, Dlg1 and Dlg4 expression levels in hippocampus following forced swim stress in mice. Sci. Rep..

[143] Krishnan M.L., Van Steenwinckel J., Schang A.L., Yan J., Arnadottir J., Le Charpentier T., Csaba Z., Dournaud P., Cipriani S., Auvynet C. (2017). Integrative genomics of microglia implicates DLG4 (PSD95) in the white matter development of preterm infants. Nat. Commun..

[144] Friedrich T., Tavraz N.N., Junghans C., Schmitz B., Döring F., Koenderink J.B., Bamberg E., Jentsch T.J., Pusch M., Decher N. (2016). A de novo mutation in ATP1A2 causes familial hemiplegic migraine type 2 in a child with alternating hemiplegia. Front. Physiol..

[145] Bozon B., Davis S., Laroche S. (2003). A Requirement for the Immediate Early Gene Zif268 in Reconsolidation of Recognition Memory after Retrieval. Neuron.

[146] Jones M.W., Errington M.L., French P.J., Fine A., Bliss T.V.P., Garel S., Charnay P., Bozon B., Laroche S., Davis S. (2001). A Requirement for the Immediate Early Gene Zif268 in the Expression of Late LTP and Long-Term Memories. Nat. Neurosci..

[147] Bristot G., Feiten J.G., Pfaffenseller B., Hizo G.H., Possebon G.M.P., Valiati F.E., Pinto J.V., Caldieraro M.A., Fleck M.P.D.A., Gama C.S. (2024). Early Growth Response 1 (EGR1) Is Downregulated in Peripheral Blood from Patients with Major Psychiatric Disorders. Trends Psychiatry Psychother..

[148] Duclot F., Kabbaj M. (2017). The Role of Early Growth Response 1 (EGR1) in Brain Plasticity and Neuropsychiatric Disorders. Front. Behav. Neurosci..

[149] Gallo F.T., Katche C., Morici J.F., Medina J.H., Weisstaub N.V. (2018). Immediate Early Genes, Memory and Psychiatric Disorders: Focus on c-Fos, Egr1 and Arc. Front. Behav. Neurosci..

[150] Kammermeier P.J., Worley P.F. (2007). Homer 1a uncouples metabotropic glutamate receptor 5 from postsynaptic effectors. Proc. Natl. Acad. Sci. USA.

[151] Wagner K.V., Hartmann J., Mangold K., Wang X.-D., Labermaier C., Liebl C., Wolf M., Gassen N.C., Holsboer F., Rein T. (2013). Homer1 Mediates Acute Stress-Induced Cognitive Deficits in the Dorsal Hippocampus. J. Neurosci..

[152] Rietschel M., Mattheisen M., Frank J., Treutlein J., Degenhardt F., Breuer R., Steffens M., Mier D., Esslinger C., Walter H. (2010). Genome-Wide Association-, Replication-, and Neuroimaging Study Implicates HOMER1 in the Etiology of Major Depression. Biol. Psychiatry.

[153] Lin X., Zhou L., Zhong J., Zhong L., Zhang R., Kang T., Wu Y. (2022). RNA-Binding Protein RBM28 Can Translocate from the Nucleolus to the Nucleoplasm to Inhibit the Transcriptional Activity of P53. J. Biol. Chem..

[154] Griñán-Ferré C., Jarne-Ferrer J., Bellver-Sanchis A., Ribalta-Vilella M., Barroso E., Salvador J.M., Jurado-Aguilar J., Palomer X., Vázquez-Carrera M., Pallàs M. (2024). Deletion of Gadd45a Expression in Mice Leads to Cognitive and Synaptic Impairment Associated with Alzheimer’s Disease Hallmarks. Int. J. Mol. Sci..

[155] You W., Xu Z., Sun Y., Valencak T.G., Wang Y., Shan T. (2020). GADD45α Drives Brown Adipose Tissue Formation Through Upregulating PPARγ in Mice. Cell Death Dis..

[156] Chen P., Zhou Z., Yao X., Pang S., Liu M., Jiang W., Jiang J., Zhang Q. (2017). Capping Enzyme mRNA-Cap/RNGTT Regulates Hedgehog Pathway Activity by Antagonizing Protein Kinase A. Sci. Rep..

[157] Majd S., Power J.H.T. (2018). Oxidative Stress and Decreased Mitochondrial Superoxide Dismutase 2 and Peroxiredoxins 1 and 4 Based Mechanism of Concurrent Activation of AMPK and mTOR in Alzheimer’s Disease. Curr. Alzheimer Res..

[158] Yan Y., Wladyka C., Fujii J., Sockanathan S. (2015). Prdx4 Is a Compartment-Specific H_2_O_2_ Sensor That Regulates Neurogenesis by Controlling Surface Expression of GDE2. Nat. Commun..

[159] Jia W., Chen P., Cheng Y. (2019). PRDX4 and Its Roles in Various Cancers. Technol. Cancer Res. Treat..

[160] Liu Y., Song H., Zhou Y., Ma X., Xu J., Yu Z., Chen L. (2021). The Oncogenic Role of Protein Kinase D3 in Cancer. J. Cancer.

[161] Ueda Y., Ooshio I., Fusamae Y., Kitae K., Kawaguchi M., Jingushi K., Hase H., Harada K., Hirata K., Tsujikawa K. (2017). AlkB Homolog 3-Mediated tRNA Demethylation Promotes Protein Synthesis in Cancer Cells. Sci. Rep..

[162] Liefke R., Windhof-Jaidhauser I.M., Gaedcke J., Salinas-Riester G., Wu F., Ghadimi M., Dango S. (2015). The Oxidative Demethylase ALKBH3 Marks Hyperactive Gene Promoters in Human Cancer Cells. Genome Med..

[163] Tasaki M., Shimada K., Kimura H., Tsujikawa K., Konishi N. (2011). ALKBH3, a Human AlkB Homologue, Contributes to Cell Survival in Human Non-Small-Cell Lung Cancer. Br. J. Cancer.

[164] Funk M.C., Bera A.N., Menchen T., Kuales G., Thriene K., Lienkamp S.S., Dengjel J., Omran H., Frank M., Arnold S.J. (2015). Cyclin O. (Ccno) Functions during Deuterosome–mediated Centriole Amplification of Multiciliated Cells. EMBO J..

[165] Peng Y., Liu J., Sun L., Zheng Q., Cao C., Ding W., Yang S., Ma L., Zhang W. (2024). GALNT9 Enrichment Attenuates MPP^+^-Induced Cytotoxicity by Ameliorating Protein Aggregations Containing α-Synuclein and Mitochondrial Dysfunction. Biol. Direct.

[166] Yang H., Sasaki T., Minoshima S., Shimizu N. (2007). Identification of Three Novel Proteins (SGSM1, 2, 3) Which Modulate Small G Protein (RAP and RAB)-Mediated Signaling Pathway. Genomics.

[167] Li J., Wang J., Ding Y., Zhao J., Wang W. (2022). Prognostic Biomarker SGSM1 and Its Correlation with Immune Infiltration in Gliomas. BMC Cancer.

[168] Kim M., de la Peña J.B., Cheong J.H., Kim H.J. (2018). Neurobiological Functions of the Period Circadian Clock 2 Gene, Per2. Biomol. Ther..

[169] Lavebratt C., Sjöholm L.K., Partonen T., Schalling M., Forsell Y. (2010). PER2 Variantion Is Associated with Depression Vulnerability. Am. J. Med. Genet. Part. B Neuropsychiatr. Genet. Off. Publ. Int. Soc. Psychiatr. Genet..

[170] Liu J.J., Sudic Hukic D., Forsell Y., Schalling M., Ösby U., Lavebratt C. (2015). Depression-Associated ARNTL and PER2 Genetic Variants in Psychotic Disorders. Chronobiol. Int..

[171] Nakamura F., Ohshima T., Goshima Y. (2020). Collapsin Response Mediator Proteins: Their Biological Functions and Pathophysiology in Neuronal Development and Regeneration. Front. Cell. Neurosci..

[172] Ravindran E., Arashiki N., Becker L.-L., Takizawa K., Lévy J., Rambaud T., Makridis K.L., Goshima Y., Li N., Vreeburg M. (2022). Monoallelic CRMP1 Gene Variants Cause Neurodevelopmental Disorder. eLife.

[173] Yoshitane H., Asano Y., Sagami A., Sakai S., Suzuki Y., Okamura H., Iwasaki W., Ozaki H., Fukada Y. (2019). Functional D-Box Sequences Reset the Circadian Clock and Drive mRNA Rhythms. Commun. Biol..

[174] Takahashi J.S. (2017). Transcriptional Architecture of the Mammalian Circadian Clock. Nat. Rev. Genet..

[175] Ideker T., Thorsson V., Ranish J.A., Christmas R., Buhler J., Eng J.K., Bumgarner R., Goodlett D.R., Aebersold R., Hood L. (2001). Integrated Genomic and Proteomic Analyses of a Systematically Perturbed Metabolic Network. Science.

[176] Oleksiak M.F., Roach J.L., Crawford D.L. (2005). Natural Variation in Cardiac Metabolism and Gene Expression in Fundulus Heteroclitus. Nat. Genet..

[177] Bigler J., Rand H.A., Kerkof K., Timour M., Russell C.B. (2013). Cross-Study Homogeneity of Psoriasis Gene Expression in Skin across a Large Expression Range. PLoS ONE.

[178] Keren L., Hausser J., Lotan-Pompan M., Vainberg Slutskin I., Alisar H., Kaminski S., Weinberger A., Alon U., Milo R., Segal E. (2016). Massively Parallel Interrogation of the Effects of Gene Expression Levels on Fitness. Cell.

[179] Strekalova T., Moskvin O., Jain A.Y., Gorbunov N., Gorlova A., Sadovnik D., Umriukhin A., Cespuglio R., Yu W.S., Tse A.C.K. (2023). Molecular signature of excessive female aggression: Study of stressed mice with genetic inactivation of neuronal serotonin synthesis. J. Neural Transm..

[180] Strekalova T., Svirin E., Gorlova A., Sheveleva E., Burova A., Khairetdinova A., Sitdikova K., Zakharova E., Dudchenko A.M., Lyundup A. (2023). Resilience and Vulnerability to Stress-Induced Anhedonia: Unveiling Brain Gene Expression and Mitochondrial Dynamics in a Mouse Chronic Stress Depression Model. Biomolecules.

[181] Lu J., Xie L., Sylvester J., Wang J., Bai J., Baybutt R., Wang W. (2007). Different Gene Expression of Skin Tissues between Mice with Weight Controlled by Either Calorie Restriction or Physical Exercise. Exp. Biol. Med..

[182] Magri C., Giacopuzzi E., Sacco C., Bocchio-Chiavetto L., Minelli A., Gennarelli M. (2021). Alterations observed in the interferon α and β signaling pathway in MDD patients are marginally influenced by cis-acting alleles. Sci. Rep..

[183] Zhang Y., Liu Z., Cao L., He Y., Ji X., Lin H., Geng C.L., Liu L., Qu P. (2017). Cold-inducible RNA-binding protein CIRBP promotes neuroinflammation via activating NF-κB and inducing IL-6 in microglial cells. Neurosci. Bull..

[184] Milenkovic V.M., Stanton E.H., Nothdurfter C., Rupprecht R., Wetzel C.H. (2019). The Role of Chemokines in the Pathophysiology of Major Depressive Disorder. Int. J. Mol. Sci..

[185] Lee J.H., Kim C.H., Kim D.G., Ahn Y.S. (2009). Microarray analysis of differentially expressed genes in the brains of tubby mice. Korean J. Physiol. Pharmacol..

[186] Liu W., Li W., Cai X., Yang Z., Li H., Su X., Song M., Zhou D.-S., Li X., Zhang C. (2020). Identification of a functional human-unique 351-bp Alu insertion polymorphism associated with major depressive disorder in the 1p31.1 GWAS risk loci. Neuropsychopharmacology.

[187] Cline B.H., Steinbusch H.W.M., Malin D., Revishchin A.V., Pavlova G.V., Cespuglio R., Strekalova T. (2012). The neuronal insulin sensitizer dicholine succinate reduces stress-induced depressive traits and memory deficit: Possible role of insulin-like growth factor 2. BMC Neurosci..

[188] Guarch J., Marcos T., Salamero M., Gastó C., Blesa R. (2008). Mild cognitive impairment: A risk indicator of later dementia, or a preclinical phase of the disease?. Int. J. Geriatr. Psychiatry.

[189] Liao W., Zhang X., Shu H., Wang Z., Liu D., Zhang Z. (2017). The characteristic of cognitive dysfunction in remitted late life depression and amnestic mild cognitive impairment. Psychiatry Res..

[190] Matsumoto K., Ono K., Ouchi H., Tsushima R., Murakami Y. (2012). Social isolation stress down-regulates cortical early growth response 1 (Egr-1) expression in mice. Neurosci. Res..

[191] Xu Y., Pan J., Sun J., Ding L., Ruan L., Reed M., Yu X., Klabnik J., Lin D., Li J. (2015). Inhibition of phosphodiesterase 2 reverses impaired cognition and neuronal remodeling caused by chronic stress. Neurobiol. Aging.

[192] Besnard A., Caboche J., Laroche S. (2013). Recall and reconsolidation of contextual fear memory: Differential control by ERK and Zif268 expression dosage. PLoS ONE.

[193] Wei J., Wu X., Luo P., Yue K., Yu Y., Pu J., Zhang L., Dai S., Han D., Fei Z. (2019). Homer1a attenuates endoplasmic reticulum stress-induced mitochondrial stress after ischemic reperfusion injury by inhibiting the PERK pathway. Front. Cell Neurosci..

[194] Wu X., Luo P., Rao W., Dai S., Zhang L., Ma W., Pu J., Yu Y., Wang J., Fei Z. (2018). Homer1a attenuates hydrogen peroxide-induced oxidative damage in HT-22 cells through AMPK-dependent autophagy. Front. Neurosci..

[195] Lominac K.D., Oleson E.B., Pava M., Klugmann M., Schwarz M.K., Seeburg P.H., During M.J., Worley P.F., Kalivas P.W., Szumlinski K.K. (2005). Distinct roles for different Homer1 isoforms in behaviors and associated prefrontal cortex function. J. Neurosci..

[196] Szumlinski K.K., Lominac K.D., Kleschen M.J., Oleson E.B., Dehoff M.H., Schwartz M.K., Seeburg P.H., Worley P.F., Kalivas P.W. (2005). Behavioral and neurochemical phenotyping of Homer1 mutant mice: Possible relevance to schizophrenia. Genes Brain Behav..

[197] Grassi D., Franz H., Vezzali R., Bovio P., Heidrich S., Dehghanian F., Lagunas N., Belzung C., Krieglstein K., Vogel T. (2017). Neuronal activity, TGFβ-signaling and unpredictable chronic stress modulate transcription of Gadd45 family members and DNA methylation in the hippocampus. Cereb Cortex.

[198] Lee A., Myung S.K., Cho J.J., Jung Y.J., Yoon J.L., Kim M.Y. (2017). Night shift work and risk of depression: Meta-analysis of observational studies. J. Korean Med. Sci..

[199] Li Y., Li G., Li J., Cai X., Sun Y., Zhang B., Zhao H. (2021). Depression-like behavior is associated with lower Per2 mRNA expression in the lateral habenula of rats. Genes Brain Behav..

[200] Wang X.L., Ji Y.B., Li S.X., Serchov T. (2025). The crosstalk between CREB and PER2 mediates the transition between mania- and depression-like behavior. Neuropsychopharmacology.

[201] Somlyai G., Jancsó G., Jákli G., Vass K., Barna B., Lakic V., Gaál T. (1993). Naturally occurring deuterium is essential for the normal growth rate of cells. FEBS Lett..

[202] Somlyai G., Nagy L.I., Puskás L.G., Papp A., Kovács B.Z., Fórizs I., Czuppon G., Somlyai I. (2023). Deuterium content of the organic compounds in food has an impact on tumor growth in mice. Curr. Issues Mol. Biol..

[203] Pomytkin I., Costa-Nunes J.P., Kasatkin V., Veniaminova E., Demchenko A., Lyundup A., Lesch K., Ponomarev E.D., Strekalova T. (2018). Insulin receptor in the brain: Mechanisms of activation and the role in the CNS pathology and treatment. CNS Neurosci. Ther..

[204] van Varsseveld N., van Bunderen C., Sohl E., Comijs H., Penninx B., Lips P., Drent M. (2015). Serum insulin-like growth factor 1 and late-life depression: A population-based study. Psychoneuroendocrinology.

[205] Strekalova T., Steinbusch H.W.M. (2010). Measuring behavior in mice with chronic stress depression paradigm. Prog. Neuropsychopharmacol. Biol. Psychiatry.

[206] Schroeter C.A., Gorlova A., Sicker M., Umriukhin A., Burova A., Shulgin B., Morozov S., Costa-Nunes J.P., Strekalova T. (2025). Unveiling the Mechanisms of a Remission in Major Depressive Disorder (MDD)-like Syndrome: The Role of Hippocampal Palmitoyltransferase Expression and Stress Susceptibility. Biomolecules.

[207] Xie F., Wang J., Zhang B. (2023). RefFinder: A web-based tool for comprehensively analyzing and identifying reference genes. Funct. Integr. Genom..

[208] de Munter J., Babaevskaya D., Wolters E.C., Pavlov D., Lysikova E., Kalueff A.V., Gorlova A., Oplatchikova M., Pomytkin I.A., Proshin A. (2020). Molecular and behavioural abnormalities in the FUS-tg mice mimic frontotemporal lobar degeneration: Effects of old and new anti-inflammatory therapies. J. Cell Mol. Med..

[209] Veniaminova E., Cespuglio R., Chernukha I., Schmitt-Boehrer A.G., Morozov S., Kalueff A.V., Kuznetsova O., Anthony D.C., Lesch K.-P., Strekalova T. (2020). Metabolic, molecular, and behavioral effects of Western diet in serotonin transporter-deficient mice: Rescue by heterozygosity?. Front. Neurosci..

[210] Vignisse J., Steinbusch H.W.M., Grigoriev V., Bolkunov A., Proshin A., Bettendorff L., Bachurin S., Strekalova T. (2014). Concomitant Manipulation of Murine NMDA- and AMPA-Receptors to Produce pro-Cognitive Drug Effects in Mice. Eur. Neuropsychopharmacol..

[211] Vignisse J., Sambon M., Gorlova A., Pavlov D., Caron N., Malgrange B., Shevtsova E., Svistunov A., Anthony D.C., Markova N. (2017). Thiamine and Benfotiamine Prevent Stress-Induced Suppression of Hippocampal Neurogenesis in Mice Exposed to Predation without Affecting Brain Thiamine Diphosphate Levels. Mol. Cell Neurosci..

[212] R Core Team (2024). R: A Language and Environment for Statistical Computing.

[213] Tenenbaum D., Maintainer B. (2025). KEGGREST: Client-Side REST Access to the Kyoto Encyclopedia of Genes and Genomes (KEGG). https://bioconductor.org/packages/KEGGREST.

[214] Kanehisa M., Goto S. (2000). KEGG: Kyoto Encyclopedia of Genes and Genomes. Nucleic Acids Res..

[215] Korotkevich G., Sukhov V., Budin N., Shpak B., Artyomov M.N., Sergushichev A. (2021). Fast gene set enrichment analysis. bioRxiv.

[216] Benjamini Y., Hochberg Y. (1995). Controlling the false discovery rate: A practical and powerful approach to multiple testing. J. R. Stat. Soc..

[217] Costa-Nunes J.P., Gorlova A., Pavlov D., Cespuglio R., Gorovaya A., Proshin A., Umriukhin A., Ponomarev E.D., Kalueff A.V., Strekalova T. (2020). Ultrasound Stress Compromises the Correlates of Emotional-like States and Brain AMPAR Expression in Mice: Effects of Antioxidant and Anti-Inflammatory Herbal Treatment. Stress.

[218] Strekalova T., Anthony D.C., Dolgov O., Anokhin K., Kubatiev A., Steinbusch H.M., Schroeter C. (2013). The differential effects of chronic imipramine or citalopram administration on physiological and behavioral outcomes in naïve mice. Behav. Brain Res..

[219] Strekalova T., Radford-Smith D., Dunstan I.K., Gorlova A., Svirin E., Sheveleva E., Burova A., Morozov S., Lyundup A., Berger G. (2024). Omega-3 alleviates behavioral and molecular changes in a mouse model of stress-induced juvenile depression. Neurobiol. Stress.

[220] Wang G.J., Thayer S.A. (1996). Sequestration of glutamate-induced Ca^2+^ loads by mitochondria in cultured rat hippocampal neurons. J. Neurophysiol..

