# Supplementary material for: Transcriptomic Insights into Late-Life Depression and the Role of Environmental Drinking Water Composition: A Study on 18-Month-Old Mice
Source: Int J Mol Sci. 2025 Oct 31;26(21):10626. doi: 10.3390/ijms262110626 (PMC12607756; doi:10.3390/ijms262110626)
Supplement: Supplementary file 1 [file ijms-26-10626-s001.zip › ijms-3845850-supplementary.pdf]

Supplementary file

Deuterium analysis in CW and DDW samples

Deuterium analysis of the samples was conducted in triplicate utilizing the equilibration technique. Therefore, an aliquot of each sample was pipetted into r tubes, which contained a vial with 5% platinum on alumina. The tubes were subsequently sealed and filled with pure hydrogen gas, facilitating the complete equilibration of water with hydrogen. This procedure ensures that the deuterium enrichment of the hydrogen gas becomes proportional to that of the water. Reference standard waters were prepared in a similar manner. The analysis was executed using continuous-flow isotope ratio mass spectrometry, employing a Europa Scientific ANCA-GSL elemental analyzer (Sercon Ltd., Cheshire, UK) and a GEO 20-20 isotope ratio mass spectrometer (Sercon Ltd., Cheshire, UK). The samples were analyzed against three reference standards: IA-R054 (δ^2H V-SMOW = +4.93‰), IA-R052 (δ^2H V-SMOW = -157.12‰), and IA-R053 (δ^2H V-SMOW = -61.97‰). All three standards are traceable to the primary reference materials V-SMOW2 (Vienna Standard Mean Ocean Water) and V-SLAP2 (Vienna Standard Light Antarctic Precipitation), as distributed by the IAEA. The IA-R054 standard served as the reference for measuring the samples and other standards. The IA-R052 standard was utilized to calibrate δ^2H, while the IA-R053 standard was employed to verify the accuracy of this calibration.

Table S1

Water sample	D, ppm		δDV-SMOW, ‰	
	mean	SD	mean	SD
**DDW**	91.7	0.3	-411.3	1.9

**CW**	140.3	0.1	-99.2	0.2

Table S1. Deuterium content in water samples Deuterium content was confirmed in water samples collected by the end of the experiment on day 22. D – Deuterium; CW- control water, DDW – deuterium depleted water.

Mineral analysis in DWW samples

The mineral content in 100 ml samples of both control and deuterium-depleted water was analyzed utilizing a novAA 400 flame atomic absorption spectrophotometer (AAS) (Analytik Jena, Jena, Germany) and a GBC 908AA graphite furnace AAS system (GBC, Victoria, Australia). The analysis adhered to the EPA 600/4-91-0101 method for mineral determination. High-purity mineral standards (CertiPUR grade) from Merck Chemicals (Darmstadt, Germany) were employed for calibration and quality control.

Table S2

**Element**	**Concentration, mg/l**
Chloride, Cl^-	103.0
Hydrocarbonate, HCO^{3-}	55.8
Calcium, Ca^{2+}	38.1
Sodium, Na^+	15.3
Potassium, K^+	14.4
Magnesium, Mg^{2+}	10.4
Iodide, I^-	<10.0
Fluoride, F^-	0.5
Sulfate, SO_4^{2-}	<0.5
Bromide, Br^-	<0.1
**pH**	**7.2**

Table S2. PH and Mineral composition of CW and DDW. Mineral composition was identical in samples of control water and deuterium depleted water showing that the depletion process did not alter the mineral content.

Table S3

Indicator	Result
Total microbial count in 1 ml at 37°C	0
Total microbial count in 1 ml at 22°C	0
Total coliform bacteria, number in 100 ml	Not detected
Thermotolerant coliform bacteria, number in 100 ml	Not detected
Glucose positive coliform bacteria, number in 100 ml	Not detected
Spores of sulfite-reducing clostridia in 20 ml	Not detected
Coliphages in 1000 ml	Not detected
Pseudomonas aeruginosa in 1000 ml	Not detected

Table S3. The bacterial count in CW and DDW samples.The bacterial count in CW and DDW indicates the absence of bacterial flora, showing a lack of contamination of water used in a study.

Table S4

	**Hippocampus**				**Prefrontal cortex**			
|FC| / FDR	≥1.25	≥1.5	≥2.0	≥2.5	≥1.25	≥1.5	≥2.0	≥2.5
<0.05	323	49	7	5	624	139	32	12
<0.01	193	42	5	4	507	127	30	12
<0.001	69	27	5	3	357	110	30	12

Table S4. Number of Differentially expressed genes (DEGs) for old vs young groups revealed in the hippocampus and prefrontal cortex with different values of Fold-Change (FC) and False Discovery Rate (FDR) applied.

Table S5

Name	Forward sequence	Reverse sequence
GAPDH	5′-TGCACCACCAACTGCTTAG-3′	5′- GGATGCAGGGATGATGTTC -3′
Hba-a1	5′-ATC CTT TCC AGG GCT TCA GC′	5′-AAA CCA TGG TGC TCT CTG GG-3′
Ccl21a	5′-CATCCCGGCAATCCTGTTCT-3′	5′-CCTCTTGAGGGCTGTGTCTG-3′
Erdr1	5′-AGATGTATGTGCCACCGACC-3′	5′-TCGTGGGTGACATCACTGTG-3′
Erg1	5′-TACCTCAGTGTCGACCTCGT-3′	5′-TCTCTCGATGACCGTCACCT-3′
Per2	5′-AATGTCCAGTGAGAGCCAGC-3′	5′-CCACAGCAAACATATCCGCG-3′
Pik3r3	5′-CGGCTCGAGGATATGGCTTT-3′	5′-TGACGTTGAGGGAGTCGTTG-3′
Prdx1	5′-TGATTGGCGCTTCTGTGGAT-3′	5′-GGTGCGCTTGGGATCTGATA-3′

Table S5. Sequences of the primers used in qPCR assay

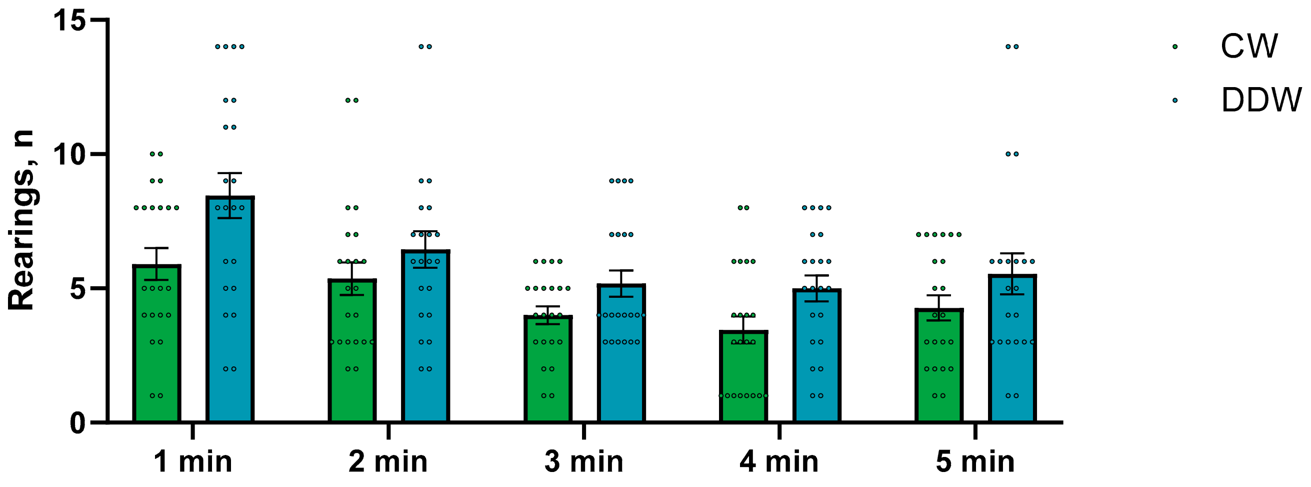

Figure S1. Rearing behavior did not differ between groups in one-minute intervals, although DDW mice showed a trend toward higher rearings score in the first minute of the test than CW-housed animals. Repeated measures two-way ANOVA. CW — control water, DDW — deuterium-depleted water. All n = 20. Bars are mean ± SEM.

O-maze and floating behaviors of CW- and DDW-mice

No significant group differences were observed in time spent in open arms, the latency to exit into the open arms (U = 170.5, p = 0.4224, Mann-Whitney test, Supplementary Fig. 2B), and the number of exits into the open arms (U = 254, p = 0.4843, Supplementary Fig. 2C) in the O-maze test. On the first day of the swim test, latency to the first floating episode did not significantly

differ between groups (U = 137.5, p = 0.0923, Mann-Whitney test, Supplementary Fig. 2D), as well as floating duration (p = 0.1820, unpaired t-test, Supplementary Fig. 2E).

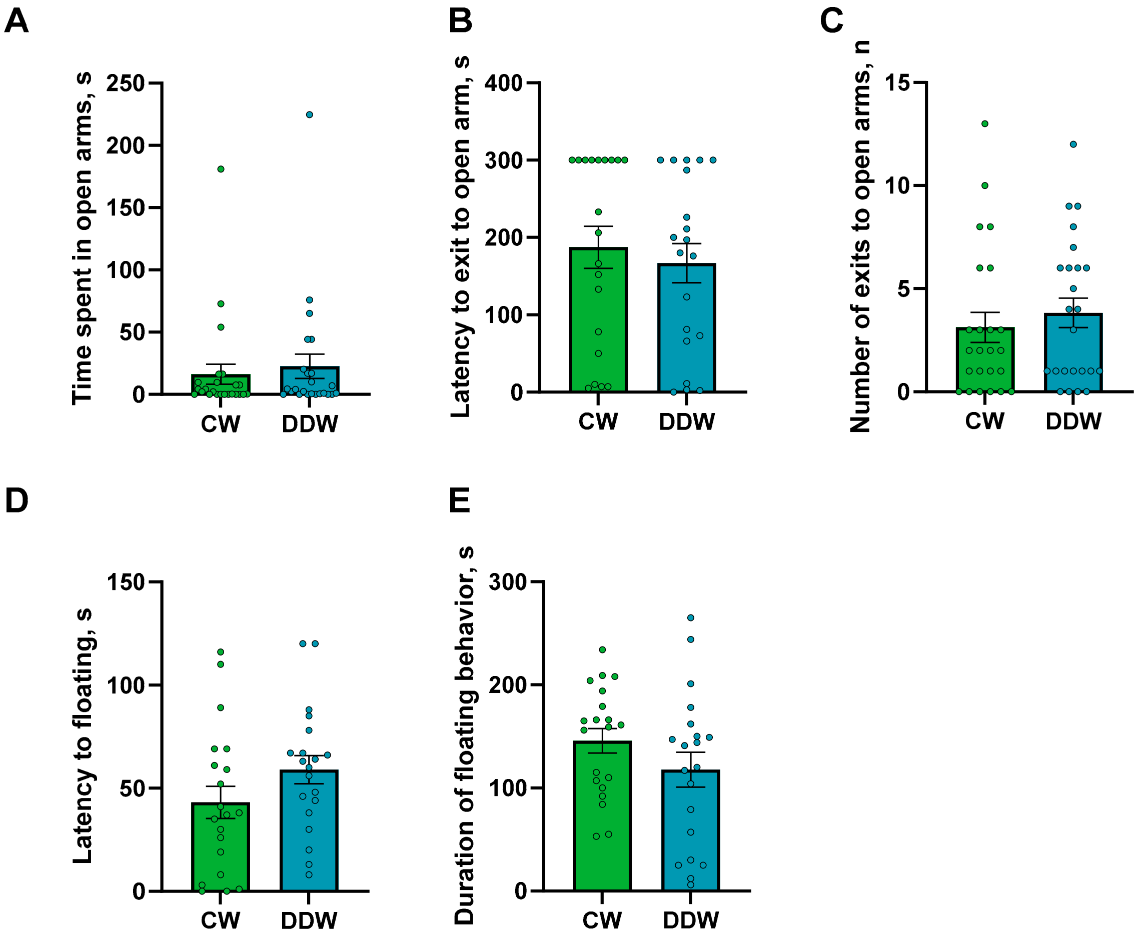

Figure S2. No significant behavioral changes observed in O-maze and on the first day of the swim test. (A) In the O-maze, no group differences in anxiety-like behaviors were observed. **(B)** No significant group differences in latency to exit to open arms and (**C**) number of exits to open arms in O-maze. (**D**) No significant differences in latency to the first floating episode and (**E**) duration of the floating behavior on the first day of the swim test. Bars are mean ± SEM. Mann-Whitney test and unpaired t-test. All n = 20. CW — control water, DDW — deuterium-depleted water.

Effects of DDW exposure on parameters of emotionality in the dark-light box in 12 m.o. mice

No significant differences between groups were shown for latency to first risk assessment (U = 13, p = 0.459, Mann-Whitney test; Supplementary Fig. 3A), total number of risk assessments (p = 0.309, unpaired t-test; Supplementary Fig. 3B), as well as for latency to exit (U = 11, p = 0.309, Mann-Whitney test; Supplementary Fig. 3C), total number of exits (U = 15, p = 0.673, Mann-Whitney test; Supplementary Fig. 3D), and total duration of exits (p = 0.065, unpaired t-test; Supplementary Fig. 3E) in the dark-light box.

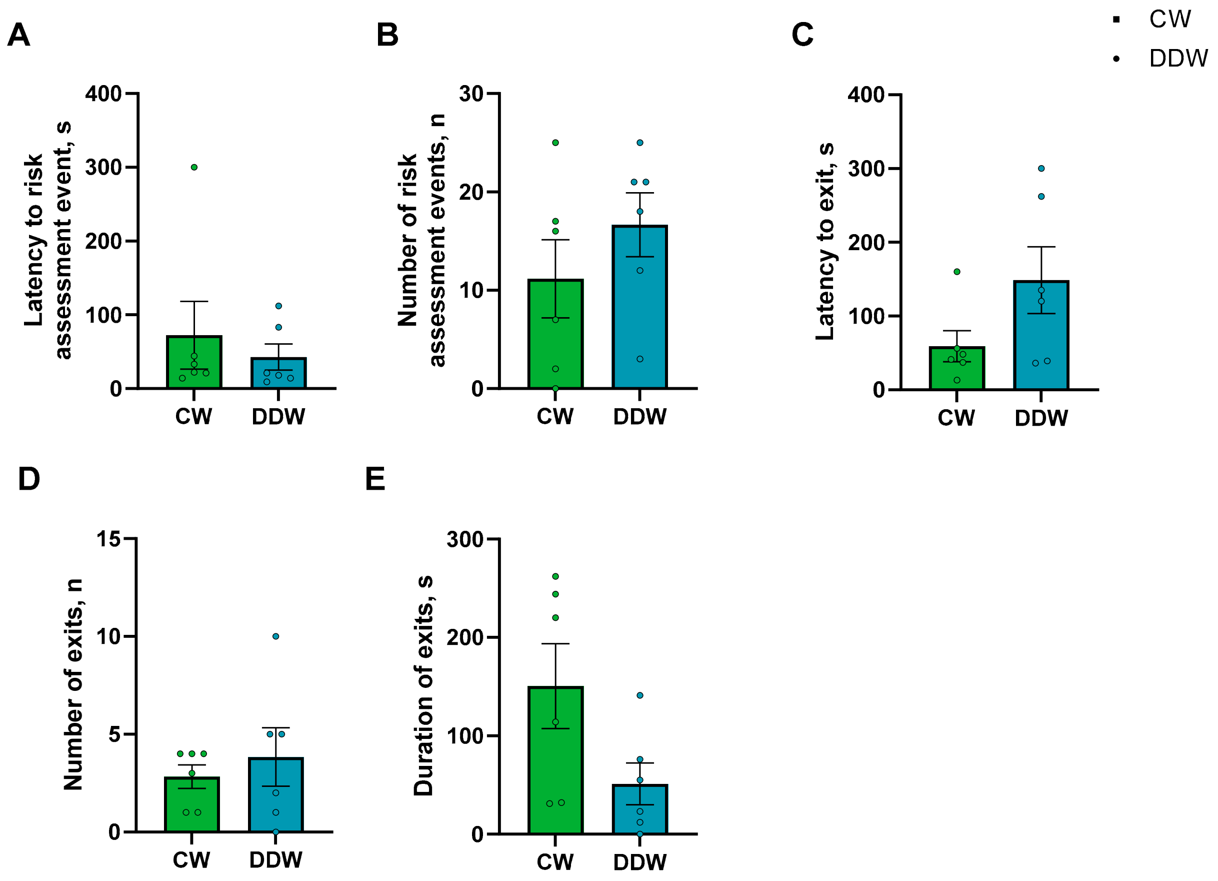

Figure S3. No significant behavioral changes observed in Dark-light box. (A) No significant differences between groups were found in latency to first risk assessment, **(B)** total number of risk assessments, **(C)** latency to exit, **(D)** total number of exits and **(E)** total duration of exits. Mann-Whitney test, or unpaired t-test; CW — control water, DDW — deuterium-depleted water. All n = 6. Bars are mean ± SEM.

qRT-PCR gene expression profiling of selected genes

qRT-PCR assay was performed on a subset of genes that showed the most prominent differential expression in the Illumina dataset or / and that were changed in their expression both in the hippocampus and prefrontal cortex. For statistical results, see ms text.

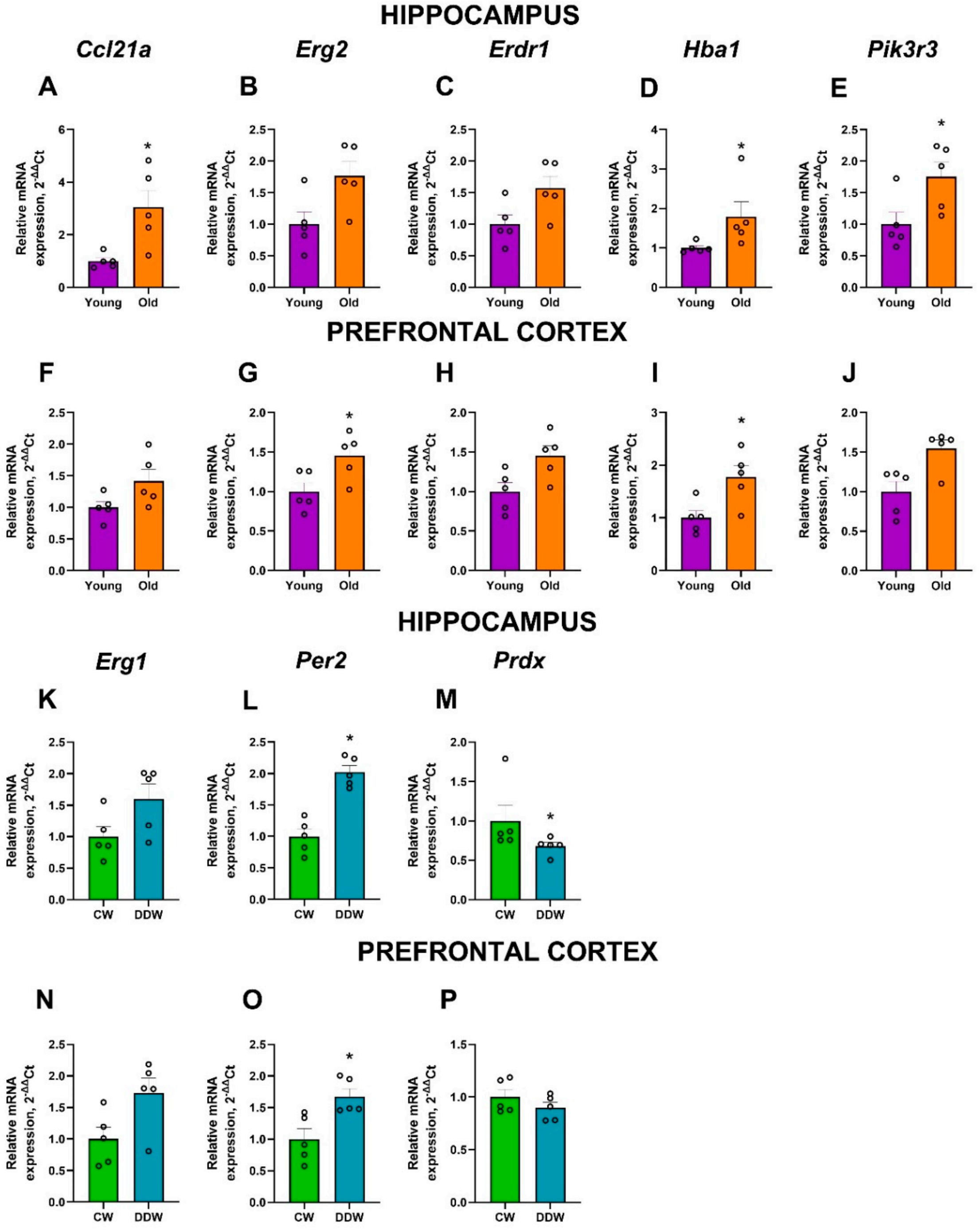

Figure S4. Differential expression of genes selected from the Illumina dataset in experimental groups of mice. **(A)** Old mice show significantly higher mRNA levels of *Ccl21a* in the hippocampus. No significant differences were observed in **(B)** *Erg2* and **(C)** *Erdr1* expression in the hippocampus. **(D)** Significant elevation in the hippocampal expression of *Hba1* and **(E)** *Pik3r3* was demonstrated in old mice compared to that in young mice. **(F)** No significant differences were observed in *Ccl21a* expression in the prefrontal cortex. **(G)** *Erg2* expression is significantly increased in the prefrontal cortex of old mice. **(H)** No significant differences were revealed in *Erdr1* expression in the prefrontal cortex. **(I)** *Hba1* expression is significantly increased in the prefrontal cortex of old mice. **(J)** No significant differences were shown in *Pik3r3* expression in the prefrontal cortex. **(K)** No significant differences were found in *Erg1* expression in the hippocampus. **(L)** *Per2* expression was significantly elevated in the hippocampus of the DDW group compared with that in the CW group. **(M)** *Prdx* expression in the hippocampus. were significantly lower in the DDW group than in the CW group. **(N)** No significant differences were found in *Erg1* expression in the prefrontal cortex. **(O)** *Per2* expression was significantly elevated in the prefrontal cortex of the DDW group compared with that the in CW group. **(P)** No significant differences were observed in *Prdx4*

expression in the prefrontal cortex. *$p<0.05$, Mann-Whitney test; CW, control water; DDW, deuterium-depleted water; $n = 5$. Bars are mean ± SEM.
